# In Silico and In Vitro Investigation of Cytotoxicity and Apoptosis of Acridine/Sulfonamide Hybrids Targeting Topoisomerases I and II

**DOI:** 10.3390/ph17111487

**Published:** 2024-11-06

**Authors:** Mohamed Badr, Elshaymaa I. Elmongy, Doaa Elkhateeb, Yasmine S. Moemen, Ashraf Khalil, Hadeer Ali, Reem Binsuwaidan, Feby Awadallah, Ibrahim El Tantawy El Sayed

**Affiliations:** 1 Department of Biochemistry, Faculty of Pharmacy, Menoufia University, Shebin El-Kom 6131567, Egypt; mohamed.badr@phrm.menofia.edu.eg; 2 Department of Pharmaceutical Chemistry, Faculty of Pharmacy, Helwan University, Ain Helwan, Cairo P.O. Box 11795, Egypt; 3 Chemistry Department, Faculty of Science, Menoufia University, Shebin El-Kom 32511, Egypt; elkhateebdoaaa@gmail.com (D.E.); yohader1@gmail.com (H.A.); feby_awadallah@yahoo.com (F.A.); ibrahimtantawy@science.menofia.edu.eg (I.E.T.E.S.); 4 Department of Clinical Biochemistry and Molecular Diagnostics, National Liver Institute, Menoufia University, Shebin El-Kom 32511, Egypt; ashkalil2010@gmail.com; 5 Clinical Pathology Department, National Liver Institute, Menoufia University, Shebin El-Kom 32511, Egypt; yasmine.moemen@gmail.com; 6 Department of Pharmaceutical Sciences, College of Pharmacy, Princess Nourah bint Abdulrahman University, P.O. Box 84428, Riyadh 11671, Saudi Arabia; rabinsuwaidan@pnu.edu.sa; 7 Clinical pathology Department, Menoufia University Hospital, Shebin El-Kom 32511, Egypt

**Keywords:** acridines, cytotoxicity, topoisomerase, molecular docking

## Abstract

Background: Sulfonamide acridine derivatives have garnered significant attention from medicinal chemists due to their diverse range of biological activities. Methods: In this study, eleven compounds were synthesized according to the literature, and their impact on cell growth inhibition, induction of apoptosis, and cell cycle distribution were assessed in three different cell lines. Their inhibitory effects on the topoisomerase (Topo) I and II were investigated in vitro. Molecular docking studies were conducted to predict the binding affinities of these compounds for crystallized downloaded topoisomerases. Results: The compounds were examined in vitro for their anticancer activity against human hepatic (HepG2) colon (HCT-8) and breast (MCF-7) carcinoma cell lines. Compound **8b** was the most active against HepG2, HCT-116, and MCF-7 with IC_50_ 14.51, 9.39, and 8.83 µM, respectively, compared to Doxorubicin as reference. In addition, it demonstrated the highest potency among the tested compounds against Topo-I, with an IC_50_ value of 3.41 µg/mL compared to the control camptothecin (IC_50_ of 1.46 μM). Compound **7c** displayed a significant inhibitory effect on Topo-II, with an IC_50_ of 7.33 μM, compared to an IC_50_ value of 6.49 μM via Doxorubicin, the control. Compounds **7c** and **8b** were assessed against topoisomerases showing induction of apoptosis and a reduction in the S phase of the cell cycle. Molecular docking demonstrated interaction with the active site as with those exhibited by the co-crystallized ligands of the crystallized proteins in both topoisomerases. Conclusion: Compounds **7c** and **8b** hold promise as potential anticancer drugs due to their anti-proliferative and proapoptotic effects, which are mediated by their action on the topoisomerase enzyme, particularly Topo II.

## 1. Introduction

**Figure 1:**
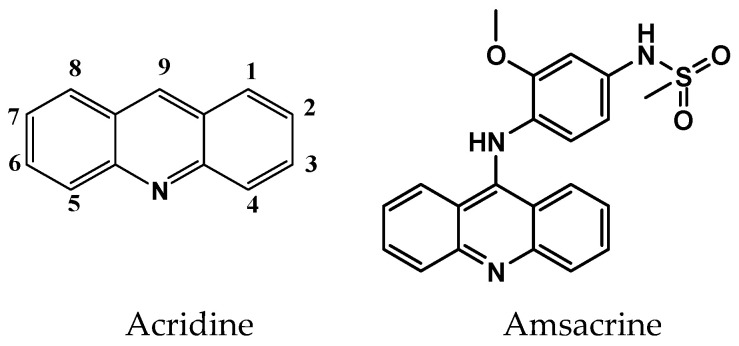
Structure of acridine-based Amsacrine (m-AMSA 8).

Topoisomerases are a crucial group of enzymes that play a vital role in various DNA-related processes, such as replication and transcription. They intricately regulate DNA structure, enabling the smooth unwinding, separation, and rejoining of strands throughout cellular processes [1]. These enzymes cleave and reconnect the DNA strands by forming a covalent bond with the DNA phosphorus group. Topoisomerases are categorized into two groups according to their mechanism of action: topoisomerases I (Topo I) and topoisomerases II (Topo-II), which are then subdivided into five subfamilies [2]. Topo-I generate temporary single-stranded DNA nicks, whereas (Topo IIα and Topo IIβ) induce temporary double-stranded DNA breaks. Cancer chemotherapy targets nuclear Topo I and Topo II, while therapeutic procedures include Top inhibitors [3,4,5]. The use of cytostatic drugs, which inhibit enzyme activity, lead to the irreversible breakage of DNA strands (via the formation of a stable DNA-topoisomerase complex), which leads to cell death. The heightened level of topoisomerase activity observed in several types of cancer has generated significant interest in the use of topoisomerase inhibitors [6]. Over the past decade, a wide variety of bioactive compounds have been produced and subjected to biological evaluation by various scientists, with the specific aim of targeting topoisomerase. These molecules can be classified into nine categories based on their structural features, and compounds that include N-heterocycles are one of these categories. Acridine derivatives are a class of heterocyclic compounds that are currently being extensively studied for their potential as anticancer drugs. Acridine derivatives, including Amsacrine (Figure 1), are part of an extensively researched group of anticancer medicines that mainly function by interfering with DNA synthesis and inhibiting or poisoning Topo- II [2,7,8,9,10,11,12,13,14]. The acridine ring is responsible for intercalating into DNA, whereas the non-interactive head group offers selectivity for the DNA–Topo cleavage complex and enhances its activity. In 1984, acridines (specifically Amsacrine) were discovered as topoisomerase II inhibitors. Since then, numerous derivatives of acridine with various substituents on the acridine chromophore have been extensively investigated as topoisomerase inhibitors [15]. Also, during the early 1980s, cytotoxic alkaloid camptothecin (CPT) was identified as a topoisomerase I inhibitor [16].

The academic literature has recorded the successful use of the hybrid pharmacophore concept, which involves combining heterocycles with established active groups such as sulfonamides, in the development of pharmaceutical drugs. Furthermore, the hybrid of thiourea and sulfonamide compounds has garnered significant interest because of their diverse range of biological effects, such as their ability to inhibit the growth of cancer cells, viruses, bacteria, parasites, and fungi [17,18,19,20,21,22]. Furthermore, the goal is to reduce drug resistance development in hybrid compounds while improving their effectiveness [23]. The combination of two bioactive chemicals via hybridization typically leads to increased activity as a result of the synergistic effects. The presence of a significant number of intramolecular hydrogen bonds between the thiourea group and the receptor pocket contributes to the enhancement of drug bioactivities [24]. In our previous study, we aimed to discover new acridine derivatives that have strong anticancer properties but cause minimal side effects. To achieve this, we synthesized and evaluated several acridine analogs with sulfonamide and thioureide side chains at the C-9 position. These analogs were prepared using nucleophilic aromatic substitution (SNAr) to replace the chlorine atom in 9-chloroacridine with either 4,4’-diaminodiphenylmethane or phenylenediamine. To make thiourea and acridine sulfonamide analogs, the 9-amino derivatives were mixed with phenyl isothiocyanate or aryl–sulfonyl chlorides. By incorporating basic side chains with para-phenylenediamine into the acridine structure at the C-9 position, we observed a significant increase in the anticancer activity of these compounds in vitro against human HepG2, colon (HCT-116), and breast (MCF-7) carcinoma cells [25]. Accordingly, this study aimed to evaluate both in silico and in vitro the inhibitory effects of the synthesized acridine analogs hybrids on both topoisomerase I and topoisomerase II, along with their antiproliferative activity on hepatocellular carcinoma cell lines.

## 2. Results

### 2.1. The Antiproliferative Activity Against HepG2, MCF7, and HCT Cell Lines

As reported in our previous study the antiproliferative activity of synthesized compounds were assessed on three cancerous cell lines, HepG2, MCF7, and HCT, using an MTT assay. Following 48 h exposure to varying concentrations of each compound, growth inhibitory curves were employed to determine the IC_50_ for each compound. These IC_50_ values were then compared to those of Doxorubicin (DOX), a known agent with powerful anti-proliferative and cytotoxic effects on these cell lines [25]. The resulting IC_50_ values for the tested compounds are tabulated below; Table 1 indicates that compounds **5b**, **8b**, **6b**, and **8a** exhibited significant anticancer activity against all three cancer cell lines. It is worth noting that **5b** and **8b** are the most active against HepG2, HCT-116, and MCF-7 with IC_50_ 8.30, 8.93, 5.88 and 14.51, 9.39, 8.83 µM, respectively, when compared to reference drug DOX used in the same assay. Dose responsive curves of antiproliferative activity for the synthesized acridine derivatives are illustrated in Figure S1.

### 2.2. Enzyme Inhibitory Assay

Based on the above-mentioned results and aiming to explore the specific inhibitory activity of the synthesized compounds, they were assessed for their potential inhibitory effect on both Topo-I and Topo-II isomerase enzymes. Subsequently, these compounds were employed to evaluate their capacity to inhibit topoisomerase activity at the established IC_50_, using supercoiled DNA as a substrate. The resulting DNA products were separated via 1% gel electrophoresis and stained with ethidium bromide. As shown in Table 2, in comparison to the control (camptothecin), which displayed an IC_50_ of 1.46 μM, compounds **6b**, **8b**, and **7d** exhibited IC_50_ values of 4.17, 3.41, and 7.67 μM, respectively, effectively reducing Topo-I activity. Similarly, compounds **7a** and **7c** displayed IC_50_ values of 8.07 and 7.33 μM, respectively, in the Topo-II assay, effectively reducing Topo-II activity, however, the reference Doxorubicin, demonstrated an IC_50_ value of 6.49 (Figure S2a,b).

### 2.3. Cell Cycle Analysis in HepG2 Cells

Compounds **8b** and **7c** were evaluated on the hepatocellular carcinoma cell line (HepG2). Compound **8b** exhibited significant topoisomerase I inhibitory activity with an IC_50_ of 3.41, while compound **7c** displayed remarkable topoisomerase II inhibitory activity with an IC_50_ of 7.33. These compounds were selected for further investigation to assess their impact on apoptosis and cell cycle arrest. As tabulated below in Table 3, the percentage of the cells in the S phase decreased from 46.39% to 39.15% when exposed to compound **8b**. Additionally, compound **7c** induced a slight reduction in the cell population in the G0–G1 phase, from 47.02% to 42.61%, and in the S phase, from 46.39% to 29.27%. This outcome suggests that compound **7c** led to cell cycle arrest in the S phase. These results exhibit a significant resemblance to the effects of the well-established anticancer drug SAHA, highlighting the substantial efficacy of the synthesized compounds.

### 2.4. Flow Cytometry for Compounds ***8b*** and ***7c*** in HepG2 Cells

**Figure 2:**
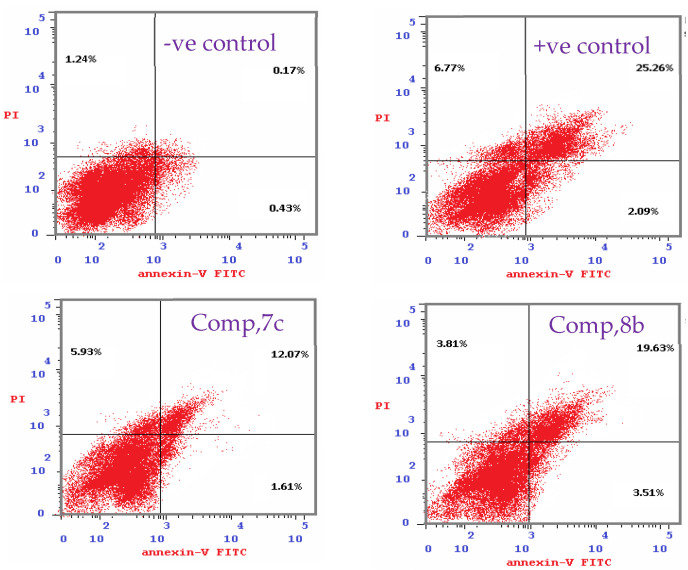
Impact of compounds **7c** and **8b** on apoptosis in HepG2 cells. HepG2 cells were treated with compounds **7c** and **8b** at the indicated concentrations for 72 h. Cells were collected, treated with Annexin V-FITC and PI, and subjected to flow cytometry as described in the text. Percentages of early and late apoptotic cells are indicated in the lower right and upper right corners, respectively.

An Annexin V-FITC assay was utilized to detect apoptosis in HepG2 cells. The cells were exposed to compounds **8b** (3.4 µM) and **7c** (7.3 µM) for 24 h, with untreated cells and cells treated with SAHA serving as negative and positive controls, respectively. After staining with propidium iodide (PI) and Annexin V-FITC, cell apoptosis was assessed via flow cytometry. The percentages of cells in the early and late apoptotic stages, as well as those undergoing necrosis, were presented in Figure 2.

Compound **7c** exhibited a 19.6% population of apoptotic cells (comprising 1.6% in the early stage and 12% in the late stage), along with 5.9% in the necrotic stage. In contrast, compound **8b** demonstrated 26.9% of the apoptotic cells (consisting of 3.5% in the early stage and 19.6% in the late stage), with 3.1% in the necrotic stage. Compound **7c** induced apoptosis to the extent of 55% and necrosis to 88% of the levels observed with SAHA, while compound **8b** induced apoptosis to the extent of 79% and necrosis to 46% of that of SAHA.

### 2.5. Selectivity Index

The higher the selectivity index of a compound to cells, the more selective the compounds are to killing or inhibiting the growth of a cancer cell compared to normal cells. Compounds with high SI values offer the potential for safer and more effective therapy in cancer therapy [26].

Accordingly, in an attempt to explore the selectivity of the prepared acridine-based derivatives to cancer cells over normal cells, both compounds were assessed for their cytotoxicity against normal cell line (THLE-2) in order to calculate the SI. Both compounds **7c** and **8b** exhibited relative cellular cytotoxicity against normal cell line THLE-2 with IC_50_ of 104 and 55.5 μM, respectively, which is better than that caused by the control anticancer drug SAHA (IC_50_ = 33.6 μM), as shown in Table 4.

Then, the selectivity index was calculated, and the results are tabulated below, in Table 5. Based on the test results, compound **7c** was found to be more selective for HePG2 and MCF-7 cells with SI > 3 than for HCT-116 cells. Meanwhile, compound **8b** was found to be selective for all the three tested cancer cells HePG2, HCT-116, and MCF-7 with SI > 3. These findings show that the synthetic compounds have the ability to target cancer cells specifically, while causing the least amount of damage to healthy cells, which is a desirable effect in chemotherapeutic treatments.

### 2.6. Molecular Docking

**Figure 3:**
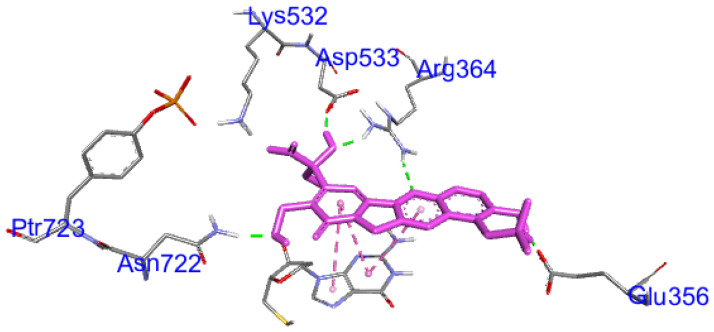
Site view of the co-crystallized ligand (represented as pink sticks) of Topo-I showing the most important active site residues, which define the receptor pocket.

**Figure 4:**
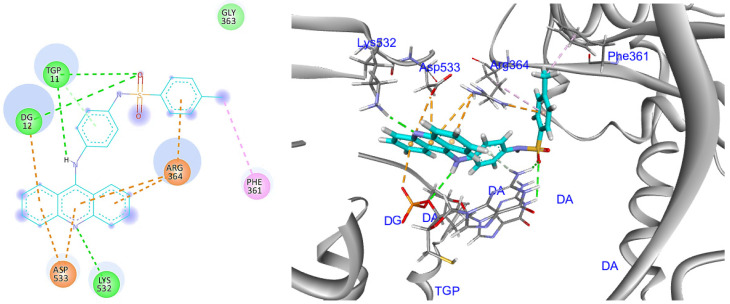
The acridine derivative **8b** docked in Topo-I ligand site (2D and 3D) showing the hydrogen bonds in a green dashed line, the hydrophobic interactions in a purple dashed line, and the pi-ion bonds in an orange dashed line.

Two proteins were downloaded from the protein data bank with pdb ID: 1K4T and 5GWK for topoisomerases-I and II, respectively. Regarding topoisomerase-I (pdb ID: 1K4T), the co-crystalized ligand downloaded with topoisomerase I—“2-(1-dimethylaminomethyl-2-hydroxy-8-hydroxymethyl-9-oxo-9,11-dihydro-indolizino [1,2-b]quinolin-7-yl)-2-hydroxy-butyric acid”, referred to as “TGG”—defined the residues in the binding pockets [27] Asp-533, Asn-722, Lys-532,Phe-361,Gly-363, and Arg-364, as shown in Figure 3. TGG was stabilized by stacking interaction with the DNA in addition to a hydrogen bond with Asp-533 and Asn-722. Residues Asp-533, Asn-722, Lys-532, and Arg-364 contributed to direct interaction with the drug. Phe-361, Arg-362, and Gly-363 were not essentially important to interact with the drug directly but were stabilizing the intercalation-binding pocket. Redocking of the co-crystallized ligand was performed for validation and the recorded RMSD value was 0.94 Å. Analyzing the resulting data upon docking compounds **6a**–**8d** revealed similar interactions to those reported by TTG, where the main amino acids involved in most of the interactions were Lys-374, Lys-532, Arg-364, Asp-533, Arg-362, and Asn-722. The binding affinities were −8.145 to −6.274 kcal/mol with perfect fitting inside the binding site, as shown in Table 6. The acridine–sulfonamide derivative **8b** recorded the least IC_50_ value against topoisomerase I (3.41 ± 0.19)µM. Analyzing the recorded scores, compound **8b** demonstrated a binding energy of −6.739 kcal/mol and the amino acids residues involved in interaction with the receptor pocket were the main residues required for direct interaction with the co-crystallized ligand including Lys-532, Asp-533, and Arg-364, in addition to Phe-361, which is important for stabilizing the intercalation binding pocket, as seen in Figure 4. There was also hydrogen bonding at the DNA binding region at TGP11 and DG12, in addition to another hydrophobic one with DG12. The docked compounds showed high affinities; the various interactions with the mentioned main residues were consistent with the recorded low IC_50_ value.

**Figure 5:**
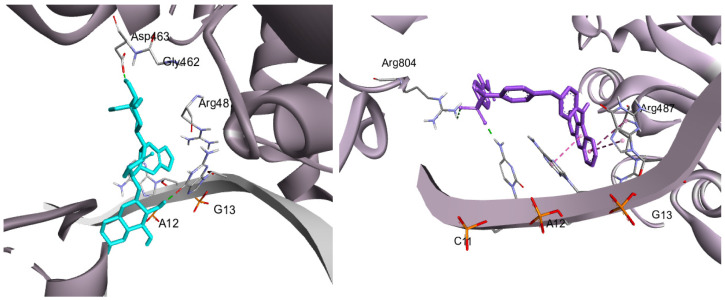
The co-crystallized ligand of the downloaded protein pdb ID; a 5GWK (in blue sticks)-defining receptor pocket and **7c** (in purple sticks) interacting with the pocket residues where hydrogen bonds are represented by a green dashed line and hydrophobic interactions are represented by a pink dashed line.

Regarding topoisomerase-IIα (pdb ID: 5GWK), the redocking of the co-crystallized ligand was performed at the ligand binding site and showed a RMSD of 1.71 Å. Upon docking the synthesized compounds at the defined ligand site, the proposed binding mode of the substituted tricyclic planar structure of the docked acridines showed affinity values ranging from −7.508 to −5.285 kcal/mol. Analyzing the docking results of the best inhibitor **7c,** which recorded the lowest IC_50_ score 7.33 ± 0.4 µM against topoisomerase II, it can be seen that it has occupied the same pocket as the co-crystallized ligand, as shown in Figure 5, forming four interactions with the pocket residues, two of which were with the terminal sulfonamide moiety, which showed hydrogen bond interaction with Arg-804 and DC11, while the planar aromatic system of substituted acridine showed hydrophobic π-π stacked and π-π T-shaped stacked interactions with DA12 and DG 13, respectively, at the DNA hinge region, as seen in Figure 5. The docking results including residues involved in the interactions as well as the types of bonding are tabulated in Table 6.

## 3. Materials and Methods

### 3.1. Chemistry

Compounds from **4**, **5a**, and **5b** were the starting materials for preparing compounds from 6a to 8d. The derivatives of acridine **6a**–**8d** were synthesized as reported in the literature, Scheme 1 [25]. (Compounds **6a**–**8d** are illustrated individually in Figure S3 and their data analysis results are illustrated in Figures S4–S16).

### 3.2. Antiproliferative Activity Assays

The antiproliferative activity of the following compounds, **6a**, **6b**, **7a**–**7e**, **8a**–**8d**, was assessed using an MTT assay. This study evaluated the cytotoxic effects of these compounds in human hepatic (HepG2), colon (HCT-116), and breast (MCF-7) carcinoma cell lines, as previously reported [25].

Moreover, the most promising compounds (**8b** and **7c**) were evaluated against normal transformed Human Liver Epithelial-2 Cell Line THLE-2, which was procured from the American Type Culture Collection. These cells were cultured in Dulbecco’s Modified Eagle’s Medium (DMEM) from Invitrogen/Life Technologies, supplemented with 10% Hyclone fetal bovine serum, 10% insulin (10 g/mL Sigma), and 1% penicillin and streptomycin. For experimental purposes, the cells were seeded in 96-well tissue culture plates at an approximate density of 5 × 10^3^ cells per well and were allowed to incubate overnight. The following day, the culture medium was replaced with fresh medium containing varying concentrations of the synthesized compounds to be tested for their toxicity. After 48 h of exposure, the medium was removed, and the cells were washed twice with phosphate-buffered saline (PBS). A new medium containing MTT solution was added, and the cells were further incubated for 4 h. The cellular viability and toxicity in response to each compound were determined using ELISA microplate reader- Bioline by measuring the absorbance at a wavelength of 450 nm.

### 3.3. Enzyme Inhibitory Assays

The compounds displaying cytotoxic activities underwent further examination for their potential as topoisomerase I and topoisomerase II inhibitors. To evaluate the catalytic activity of Topo-I, supercoiled DNA served as the substrate. Commercial samples of Topo I and supercoiled DNA were provided by TG1018-1A, Inc., TopoGEN, Inc. (Columbus, OH, USA). For the Topo-II assay, a Topo II drug kit, TopoGEN, TG1009, was obtained from the same source, with doxorubicin used as the reference drug.

#### 3.3.1. Topoisomerase I Inhibitory Assay

Camptothecin and the tested compounds were mixed with Topo-I DNA complex and incubated at 37 °C for 30 min. A subsequent increase in temperature to 65 °C for 30 min rendered the enzyme inactive. To visualize the cleavage products after incubation, we used 1% agarose gel electrophoresis to separate the samples.

#### 3.3.2. Topoisomerase II Inhibitory Assay

To assess the Topo-II activity, a typical enzyme reaction was set up using human Topo-II, supercoiled pHot1 DNA as the substrate, the test drug, and the assay buffer. After incubation at 37 °C for 30 min, the mixture underwent a chemical reaction. The reaction was halted by introducing a solution containing 10% sodium dodecyl sulfate and proteinase K and incubating it at 37 °C for 15 min. Following that, the DNA was stained and ran on a 1% agarose gel for one to two hours using a Bio-Rad gel electrophoresis apparatus. Both supercoiled and linear DNA strands were included in the gel to serve as indicators for DNA-Topo-II intercalation.

### 3.4. Cell Cycle Analysis

Approximately 1 × 10^5^ HepG2 cells were seeded into each well of a six-well plate and incubated for 24 h. Following incubation, the cells were treated with compounds **8b** and **7c** as specified for an additional 24 h. Subsequently, the cells were harvested, fixed in 70% ethanol, and incubated for 12 h at 4 °C. After fixation, the cells were centrifuged, and the cell pellets were washed and resuspended in 0.5 mL of cold phosphate-buffered saline (PBS). The cells were then stained with 5 μg/mL of propidium iodide (PI; Sigma, Panama City, Panama), and the DNA content was determined using a BD FACSCalibur flow cytometer (Becton Dickinson, Franklin Lakes, NJ, USA). Data analysis was performed using CellQuest software, and 10,000 events were analyzed for each sample.

### 3.5. Apoptosis Assay

An Annexin V-FITC assay was employed to detect apoptosis in the HepG2 cells. In a six-well plate, approximately 2 × 10^5^ cells were incubated with compounds **8b** and **7c** for 24 h. Following incubation, the cells were harvested, washed twice with cold PBS, and resuspended in 500 μL of Annexin V-FITC binding buffer (10 µM HEPES, 140 µM NaCl, and 2.5 µM CaCl_2_ at pH 7.4) and incubated for 15 min at room temperature. Subsequently, the cells were stained with 5 μL of Annexin V-FITC and incubated in the dark at 4 °C for 30 min, followed by the addition of 5 μL of propidium iodide (PI). Cell apoptosis was detected using a FACSCalibur flow cytometer (Becton-Dickinson, Franklin Lakes, NJ, USA). Data analysis was carried out using CellQuest software, and 10,000 events were analyzed for each sample. The Annexin V assay classified the cells as follows: viable cells were both Annexin V-FITC and PI negative. The cells in early apoptosis were Annexin V-FITC positive and PI negative, whereas the cells in late apoptosis were positive for both Annexin V and PI. The necrotic and dead cells were negative for Annexin V-FITC and positive for PI.

### 3.6. Selectivity Index Analysis

The selectivity index (SI) was obtained from the IC_50_ value of the compound against normal cells divided by the IC_50_ value of the cancer cells [28,29]. Accordingly, each SI value was calculated using the formula given below:

SI = IC_50_ for normal cells/IC_50_ for cancer cells.

Compounds were classified as high selectivity if the SI value was >3 and less selective if the SI value was <3 [30].

### 3.7. Molecular Docking

Molecular modeling studies were performed where two proteins were retrieved for docking from the protein data bank; one was the crystal structure topoisomerase-I along with its co-crystallized ligand (PDB ID:1K4T), while the other was topoisomerase2α protein with its co-crystallized ligand, which was downloaded with (PDB ID: 5GWK). Every compound structure was sketched as a 2D structure in a chem-sketch then converted to 3D using open babel and saved in sdf formate, which can be opened in UCSF-chimera 1.17.3 and Discovery Studio. Ligand and protein were then prepared using UCSF-chimera by adding hydrogen and assigning Gasteiger charges, then energy minimization. Grid box was generated using Auto-grid allocated at the macromolecule center. The grid box was then resized to cover the whole protein. Docking was performed using AutoDock vina. The results were saved as pdbqt and then visualized using Biovia Discovery Studio v21.1.0.20298 [31].

## 4. Conclusions

This work describes the synthesis of three novel series of acridine–sulfonamide hybrids, **6a**–**b**, **7a**–**e** and **8a**–**8d**. An MTT assay was performed against three cancer cell lines where compound **8b** recorded the best results with IC_50S_ values 14.51 ± 1.4, 9.39 ± 0.9, and 8.83 ± 0.9 µM against HepG2, HCT-116, and MCF-7 cell lines, respectively. The compounds’ capacity to inhibit topoisomerase activity was established using supercoiled DNA as a substrate where **8b** exhibited the best IC_50_ score of 3.41 µM against Topo-I, and **7c** displayed remarkable Topo-II inhibitory activity with an IC_50_ of 7.33 µM. Upon investigating their impact on apoptosis and cell cycle arrest, compound **8b** dropped the proportion of cells in the S phase from 46.39% to 39.15%. Furthermore, compound **7c** caused a minor decrease in the cell population in the S phase (from 46.39% to 29.27%), and in the G0–G1 phase (from 47.02% to 42.61%). Compound **7c** induced apoptosis to the extent of 55% and necrosis to 88% of the levels observed with SAHA as +ve control, while compound **8b** induced apoptosis to the extent of 79% and necrosis to 46% of that of SAHA. Furthermore, compounds **8b** and **7c** were evaluated on a THLE2 normal cell line using SAHA as a reference, where they exhibited cellular cytotoxicity with IC_50_ of 104 and 55.5 μM, respectively, in comparison to the control drug SAHA IC_50_ = 33.6 μM. In an attempt to explore the selectivity of the tested derivatives to cancer cells, the selectivity index was calculated for both compounds **7c** and **8b** which revealed that both the compounds demonstrated SI > 3 except for **7c** and HCT-116 cells; however, compound **8b** was more selective than **7c** for HepG2, HCT-116, and MCF-7. These findings show that the synthetic compounds have the ability to specifically target cancer cells while causing less damage to healthy cells. Molecular modeling studies revealed that some amino acids residues involved in the interaction of the tested compounds **8b** and **7c** with the receptor pocket were the main residues required for interaction with the co-crystallized ligand as well. Overall, compounds **7c** and **8b** can be considered as promising candidates for further anticancer drug development.

## Figures and Tables

**Scheme 1:**
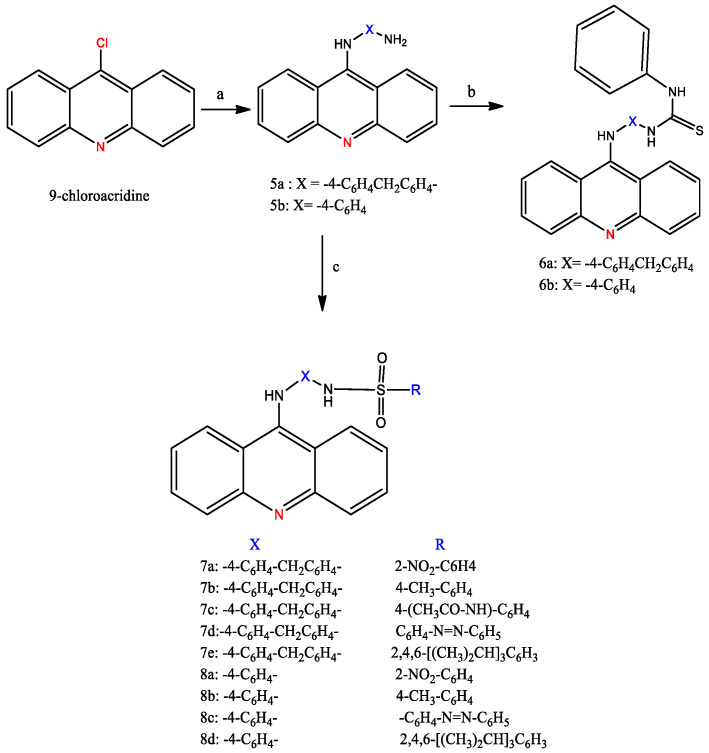
Synthesis of acridine amalogues: (a) Appropriate amine, H_2_N-X-NH_2_, EtOH, Er3N, Reflux (below 80 °C), 2.5–7 h. (b) Chiloroform, Ph-N=C=S, stirring at room temperature, (2–8 day). (c) ArSO_2_Cl, DMF, Et3N, (rt. or reflux).

**Table 1:** In vitro cytotoxicity of the tested hybrids against three cancer cell lines.

Compounds	IC_50_ (µM)		
	HepG-2	HCT-116	MCF-7
**5b**	8.30 ± 0.7	8.93 ± 0.8	5.88 ± 0.4
**6a**	36.71 ± 3.5	47.21 ± 3.9	40.93 ± 3.8
**6b**	17.98 ± 1.7	13.07 ± 1.2	16.47 ± 1.5
**7a**	62.32 ± 4.4	72.16 ± 4.7	88.36 ± 5.1
**7b**	93.60 ± 5.3	88.53 ± 4.8	73.81 ± 4.5
**7c**	31.07 ± 3.1	40.61 ± 3.7	29.08 ± 2.7
**7d**	45.49 ± 3.9	57.96 ± 4.3	33.65 ± 3.3
**7e**	55.35 ± 4.2	66.67 ± 4.5	50.76 ± 4.0
**8a**	20.63 ± 1.8	15.18 ± 1.6	10.54 ± 1.0
**8b**	14.51 ± 1.4	9.39 ± 0.9	8.83 ± 0.9
**8c**	22.38 ± 2.0	35.69 ± 3.4	25.47 ± 2.4
**8d**	23.13 ± 2.2	29.85 ± 2.8	19.69 ± 1.7
**DOX**	4.50 ± 0.2	5.23 ± 0.3	4.17 ± 0.2

**Table 2:** Potential inhibitory effect of compounds (**6a**–**8d**) on both Topo I and Topo II activities reflected by their IC_50_ µM.

Compound	Topo-I (IC_50_) µM	Topo-II (IC_50_) µM
**6a**	27.6 ± 1.5	101 ± 5.51
**6b**	4.17 ± 0.23	24.5 ± 1.34
**7a**	18.4 ± 1	8.07 ± 0.44
**7b**	82.5 ± 4.49	234 ± 12.7
**7c**	16.6 ± 0.9	7.33 ± 0.4
**7d**	7.67 ± 0.42	21.6 ± 1.18
**7e**	42.2 ± 2.29	38.7 ± 2.1
**8a**	15 ± 0.81	10.9 ± 0.59
**8b**	3.41 ± 0.19	16.2 ± 0.88
**8c**	21.4 ± 1.17	22 ± 1.2
**8d**	11.9 ± 0.65	32.7 ± 1.78
Cisplatin	5.71 ± 0.31	11.3 ± 0.62
Doxorubicin	3.36± 0.18	6.49 ± 0.35
Camptothecin	1.64 ± 0.09	-

**Table 3:** Cell cycle analysis revealing DNA content percentage by control, compound **7c** and compound **8b**.

Code	G0–G1	%S	%G2/M	%Pre-G1
**7c**/HepG2	42.61	29.27	28.12	19.61
**8b**/HepG2	51.19	39.15	9.66	26.95
SAHA/HepG2	47.02	46.39	6.59	34.12
cont. HepG2	44.06	35.11	20.83	1.84

**Table 4:** In vitro cytotoxicity of compounds **7c** and **8b** against THLE-2 cell line.

Compound	IC_50_ µM
**7c**	104 ± 5.86
**8b**	55.5 ± 3.13
SAHA	33.6 ± 1.89

**Table 5:** Selectivity index of compounds **7c** and **8b** on HePG2, HCT-116, and MCF-7 cell lines.

Compound	HePG2		HCT-116		MCF-7		THLE-2
	IC_50_ µM	SI	IC_50_ µM	SI	IC_50_ µM	SI	IC_50_ µM
**7c**	31.07 ± 3.1	3.35	40.61 ± 3.7	2.56	29.08 ± 2.7	3.57	104 ± 5.86
**8b**	14.51 ± 1.4	3.82	9.39 ± 0.9	5.91	8.83 ± 0.9	6.28	55.5 ± 3.13

**Table 6:** Docking results for compounds **6a**–**8d** at the downloaded proteins topoisomerase I and II.

Compound	Energy (kcal/mol)	Amino Acids (Ligand Interaction Type)	Energy (kcal/mol)	Aminoacids (Ligand Interaction Type)
**6a**	−7.90	Lys-374 (H-bond acceptor) Lys-532 (H-bond acceptor) Arg-364 (pi-cation) Arg-364 (pi-cation)	−6.45	His-759 (H-bond acceptor) Lys-489 (pi-alkyl) DA 12 (pi-pi) DA 12 (pi-pi)
**6b**	−6.52	Arg-364 (H-bond acceptor) TGP 11 (pi-pi)	−5.99	DG 13 (H-bond donor) DC 11 (pi-alkyl) DA 12 (pi-pi)
**7a**	−7.21	Asp-533 (H-bond donor) Arg-364 (H-bond acceptor) Arg-364 (H-bond acceptor) Arg-364 (H-bond acceptor)	−6.43	Met-766 (H-bond donor) DA 12 (pi-pi)
**7b**	−6.76	Thr-718 (H-bond donor) Lys-532 (H-bond acceptor)	−6.46	Met-766 (H-bond donor) DA 12 (H-pi)
**7c**	−8.14	Lys-532 (H-bond acceptor) Arg-364 (pi-cation)	−6.51	DC11 (H-bond acceptor) Arg-804(H-bond acceptor) DG12 (pi-pi stacked) DG 13 (pi-pi)
**7d**	−7.84	Asn-722 (H-bond acceptor) Lys-425 (pi-alkyl) Lys-425 (pi-alkyl)	−6.96	DC 14 (H-bond donor) Glu-461(H-bond donor) DC 14 (H-bond acceptor)
**7e**	−8.15	Arg-362 (pi-alkyl) Lys-425 (pi-alkyl) DG12 (H-bond acceptor)	−7.37	-
**8a**	−6.27	Asn-722 (H-bond acceptor)	−5.75	Met-766 (H-bond donor) Met-765 (H-bond donor) DA 12 (pi-pi)
**8b**	−6.74	Lys-532 (H-bond acceptor) Arg-364 (pi-cation) TGP 11 (H-bond acceptor) DG 12 (H-bond acceptor) DG 12 (pi-alkyl) Asp-533 (pi-anion) Phe-361 (pi-alkyl)	−5.29	Asp-463 (H-bond acceptor) Ser-464 (H-bond acceptor) DA 12 (pi-pi) DA 12 (pi-pi)
**8c**	−6.88	Arg-364 (H-bond acceptor)	−6.78	DA 12 (H-bond donor) DA 12 (pi-alkyl)
**8d**	−7.49	Lys-532 (H-bond acceptor) TGP11 (pi-alkyl)	−7.51	Met-762 (H-bond donor) DG 13 (H-bond donor) DC 11 (H-bond acceptor) DA 12 (H-bond acceptor)

## Data Availability

The original contributions presented in the study are included in the article/Supplementary Material, further inquiries can be directed to the corresponding authors.

## Supplementary Materials

The following supporting information can be downloaded at: https://www.mdpi.com/article/10.3390/ph17111487/s1, Figure S1: dose responsive curve of anticancer activity for acridine; Figure S2a: inhibition power of the synthesized compounds and the control on topo I-DNA cleavage complex formation; Figure S2b: inhibition power of the synthesized compounds and the control on topo II -DNA cleavage complex formation. Figure S3: chemical structures of the synthesized compounds; Figures S4–S16: data analysis results for the synthesized compounds.
