# Supplementary material for: In Silico and In Vitro Investigation of Cytotoxicity and Apoptosis of Acridine/Sulfonamide Hybrids Targeting Topoisomerases I and II
Source: Pharmaceuticals (Basel). 2024 Nov 6;17(11):1487. doi: 10.3390/ph17111487 (PMC11597879; doi:10.3390/ph17111487)
Supplement: Supplementary file 1 [file pharmaceuticals-17-01487-s001.zip › pharmaceuticals-3252752-supplementary.pdf]

Article

In silico and in vitro investigation of cytotoxicity and apoptosis of acridine/sulfonamide hybrids targeting topoisomerases I and II

Mohamed Badr [1], Elshaymaa I. Elmongy [2,*], Doaa Elkhateeb [3,4], Yasmine S. Moemen [5], Ashraf Khalil [4], Hadeer Ali [3], Reem Binsuwaidan [6], Feby Awadallah [3,7] and Ibrahim El Tantawy El Sayed[3]

[1] Department of Biochemistry, Faculty of Pharmacy, Menoufia University, Shebin El-Kom, Egypt; mohamed.badr@phrm.menofia.edu.eg
[2] Department of Pharmaceutical Chemistry, Faculty of Pharmacy, Helwan University, Ain Helwan, Cairo P.O. Box 11795, Egypt
[3] Chemistry Department, Faculty of Science, Menoufia University, Shebin El-Kom 32511, Egypt; ibrahimtantawy@science.menofia.edu.eg (I.E.T.E.S.); yohader1@gmail.com (H.A.); feby_awadallah@yahoo.com (F.A.); elkhateebdoaaa@gmail.com (D.E.)
[4] Department of Clinical Biochemistry and Molecular Diagnostics, National Liver Institute, Menoufia University, Shebin Elkom, Egypt; ashkalil2010@gmail.com
[5] Clinical Pathology Department, National Liver Institute, Menoufia University, Shebin El-Kom, Egypt; yasmine.moemen@gmail.com
[6] Department of Pharmaceutical Sciences, College of Pharmacy, Princess Nourah bint Abdulrahman University, P.O. Box 84428, Riyadh 11671, Saudi Arabia; rabinsuwaidan@pnu.edu.sa
[7] Clinical pathology Department, Menoufia University Hospital, Shebin El Koom, Egypt
* Correspondence: shaymaa.taha@pharm.helwan.edu.eg

Figure S1: Dose responsive curve of anticancer activity for acridine derivatives (5a-7i)

According to lab numbering :Compound 7f refers to compound 8a, Compound 7g refers to compound 8b, Compound 7h refers to compound 8c, Compound 7i refers to compound 8d

Dose responsive curve of anticancer activity for acridine derivatives

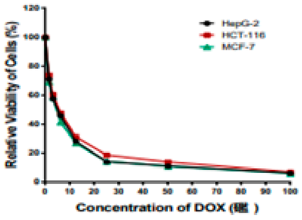

Fig. 1: Cytotoxicity of Dox

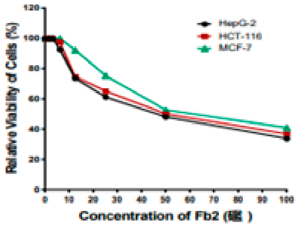

Fig. 2: Cytotoxicity of Compound **5a**

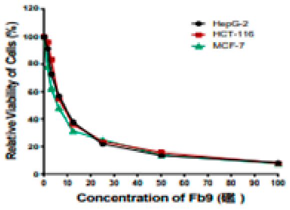

Fig. 3: Cytotoxicity of compound **5b**

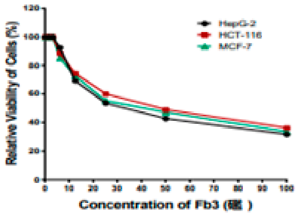

Fig. 4: Cytotoxicity activity of Compound **6a**

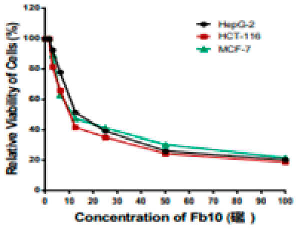

Fig. 5: Cytotoxicity of Compound **6b**

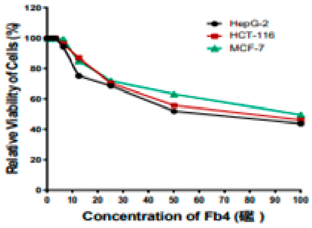

Fig. 6: Cytotoxicity activity of Compound **7a**

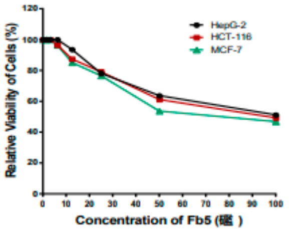

Fig. 7: Cytotoxicity of Compound **7b**

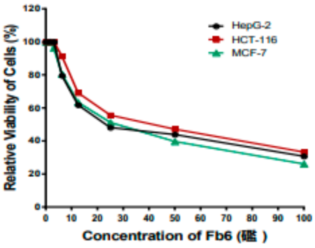

Fig. 8: Cytotoxicity activity of Compound **7c**

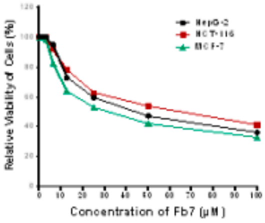

Fig. 9: Cytotoxicity of Compound **7d**

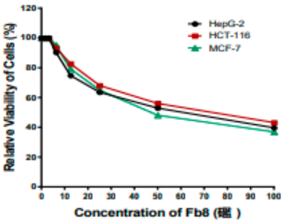

Fig. 10: Cytotoxicity of Compound **7e**

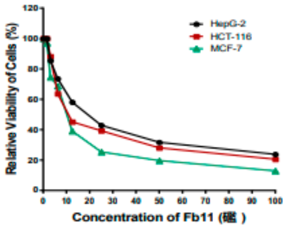

Fig. 11: Cytotoxicity of Compound **7f**

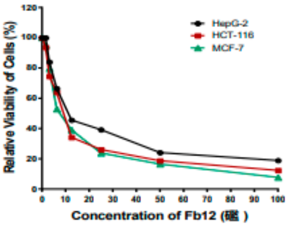

Fig. 12: Cytotoxicity of Compound **7g**

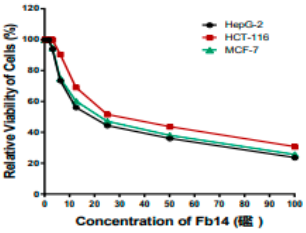

Fig. 13: Cytotoxicity of Compound **7h**

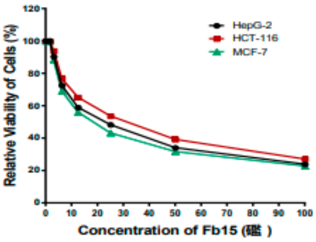

Fig. 14: Cytotoxicity of Compound **7i**

Figure S2a: Diagrammatic representation reveals the inhibition power of the synthesized compounds and the control on topo I-DNA cleavage complex formation.

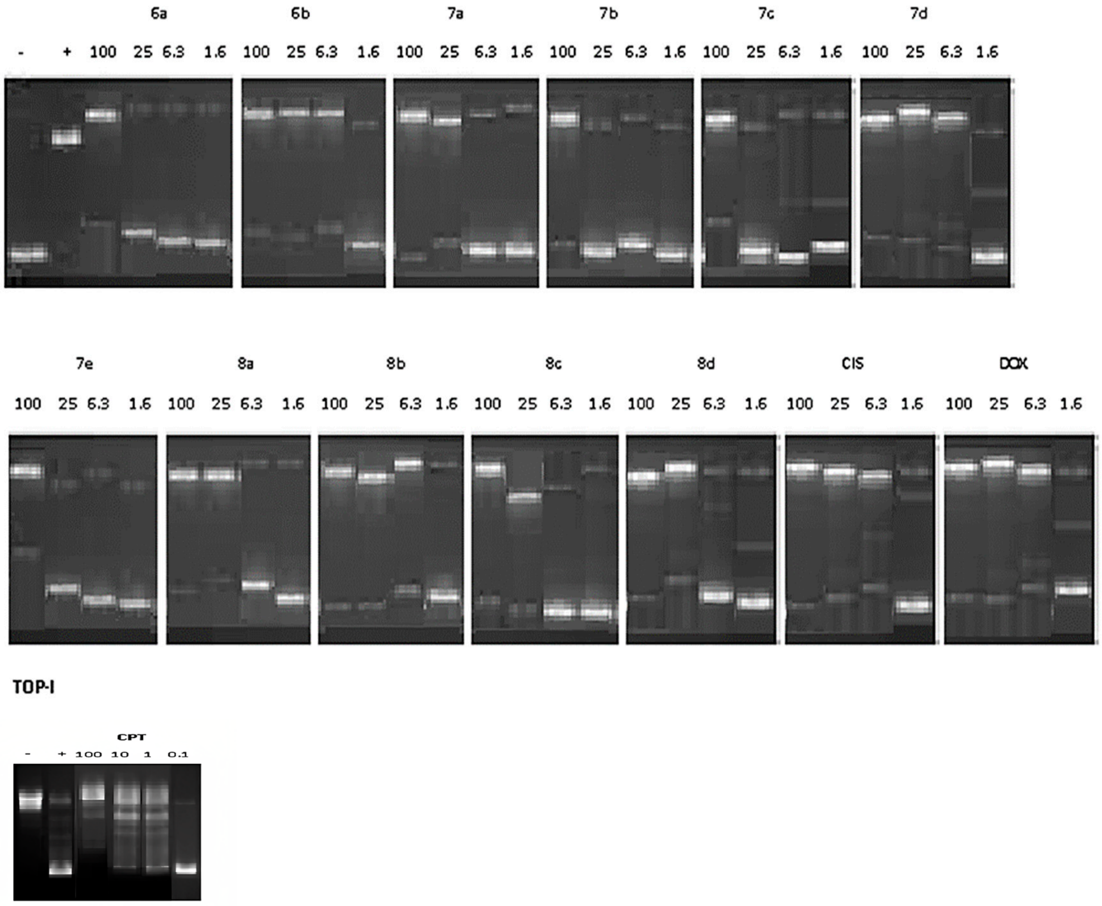

Figure S2 b: Diagrammatic representation reveals the inhibition power of the synthesized compounds and the control (Doxorubicin) on topo II-DNA cleavage complex formation.

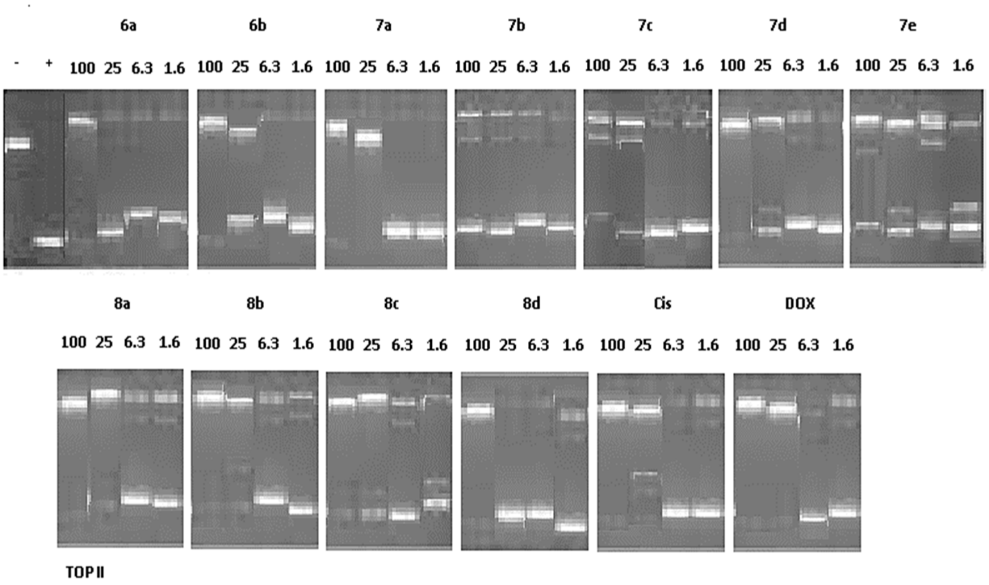

Figure S3: Chemical Structures of the synthesized compounds

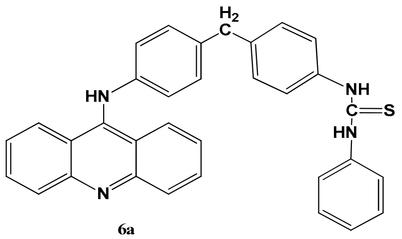

6a

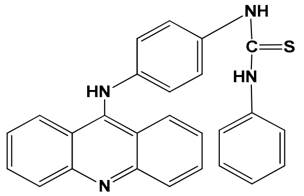

6b

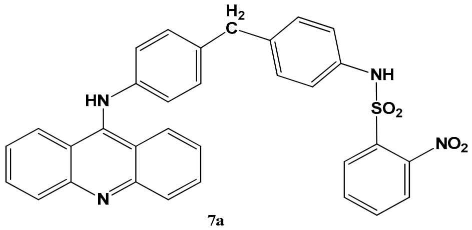

7a

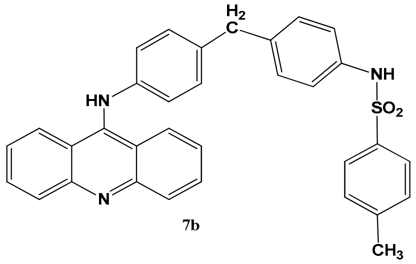

7b

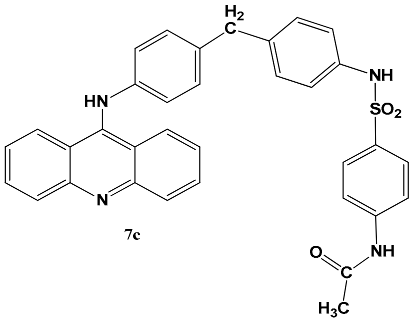

7c

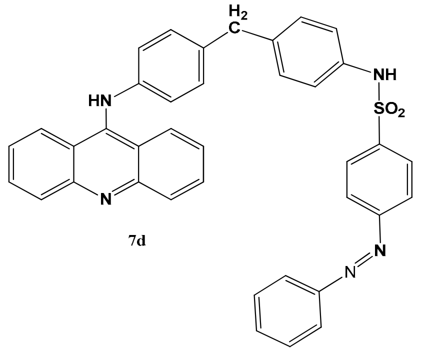

7d

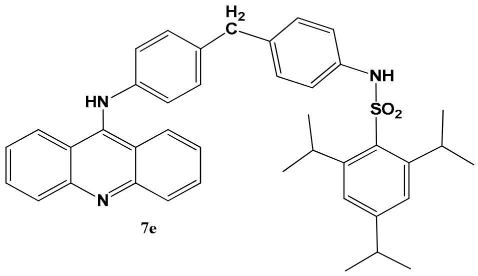

7e

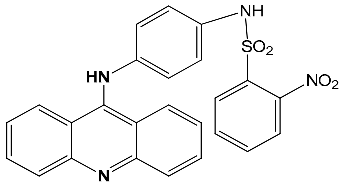

8a

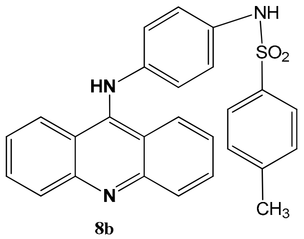

8b

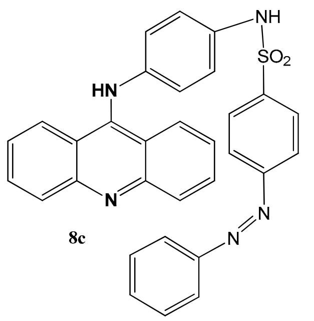

8c

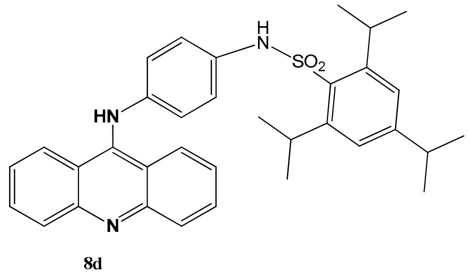

8d

Data Analysis Results for The Synthesized Compounds.

1- **9. Chloroacridine**

IR (KBr) cm-1: 3434 (NH), 3073 (CH-Ar), 1626(C=N), 1547 (C=CforAr), 1271 (C-N). ^{1}H NMR (CDCl3, 300 MHz), δ(ppm): 7.66 – 7.71 (m, 2H, Ar-H), 7.84 – 7.89 (m, 2H, Ar-H), 8.31 – 8.34 (d, J=8.4 Hz, 2H, Ar-H), 8.46 – 8.49 (d, J=8.7Hz, 2H, Ar-H).

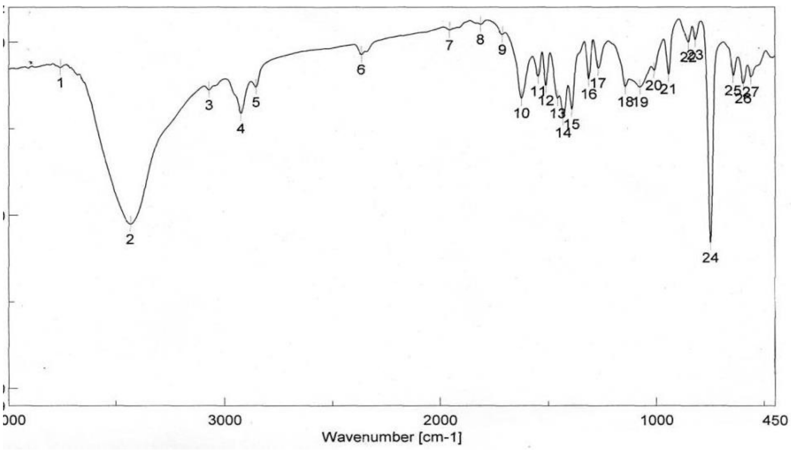

Figure S4a: IR spectrum of the compound in KBr.

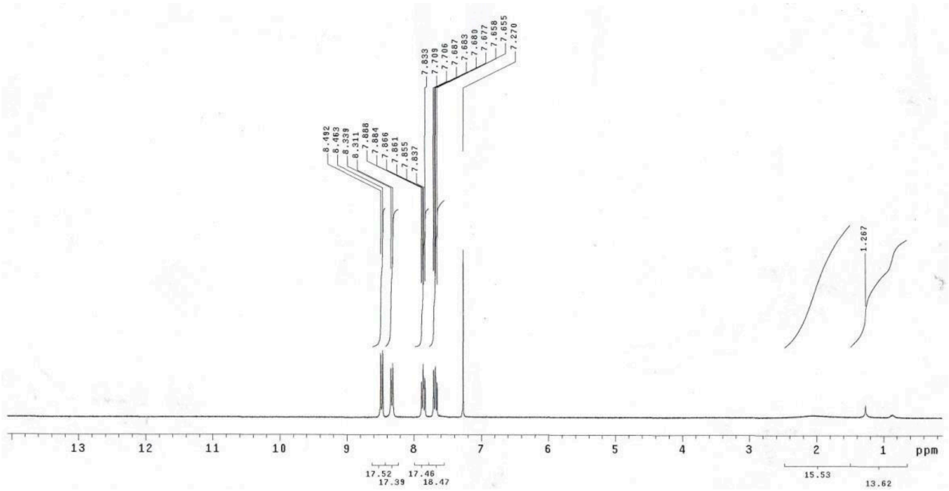

Figure S4b: ^{1}HNMR spectrum of compound (4) in chloroform

2- **N – (4-(4-Aminobenzyl)phenyl)acridin-9-amine(5a):** Yield (0.65gm, 93%) as an orange solid, m.p. 202°C, IR (KBr) cm-1: 3392(NH2+NH) overlap, 3020(CH- Ar), 2923(CH- Alkane), 1619 (C=N), 1587(C=C – Ar), 1361(C-N, Aromatic amine), 1259(=C-N).^{1}H – NMR (CDCl3, 300 MHz), δ(ppm):3.56 (br.s, 2H, NH2), 3.84 (br.s, 2H, CH2), 6.61–6.84 (m, 4H, Ar-H), 6.952–7.230 (m, 4H, Ar-H), 7.66 (br.s, 1H, NH-Ar), 8.01–8.04 (m, 8H, Ar-H). EIMS, m/z (C26H21N3) calcd.,375.465 [M]+; found,375.11.

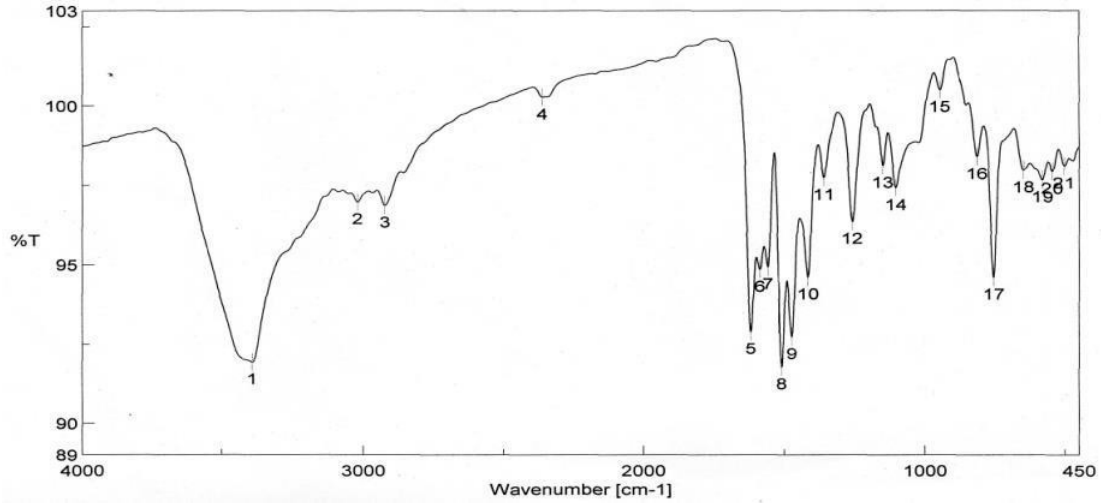

Figure S5a:IR Spectrum of compound (5a) in KBr

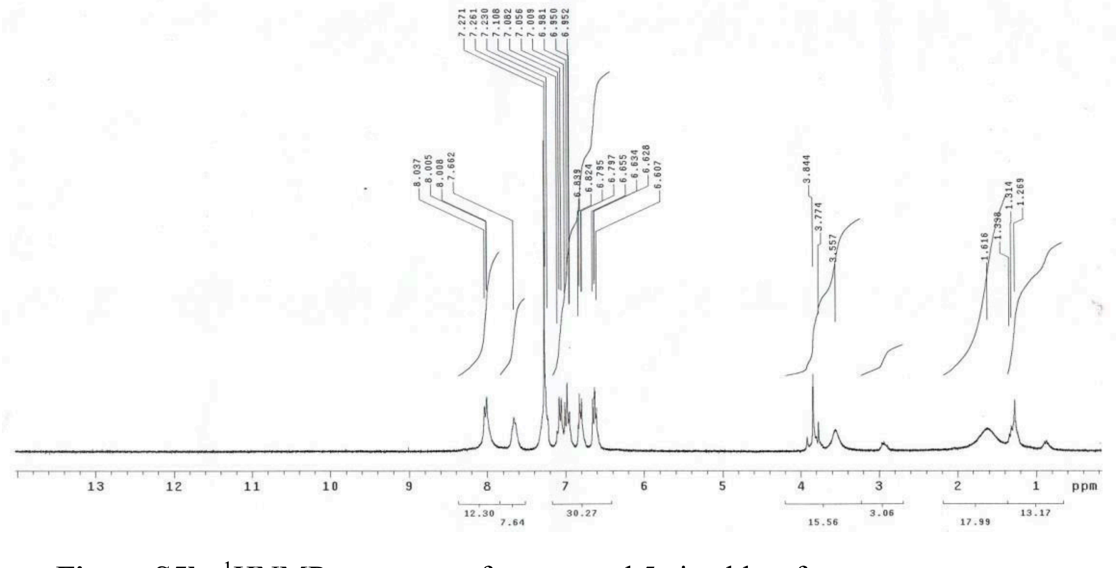

Figure S5b: ^{1}HNMR spectrum of compound 5a in chloroform

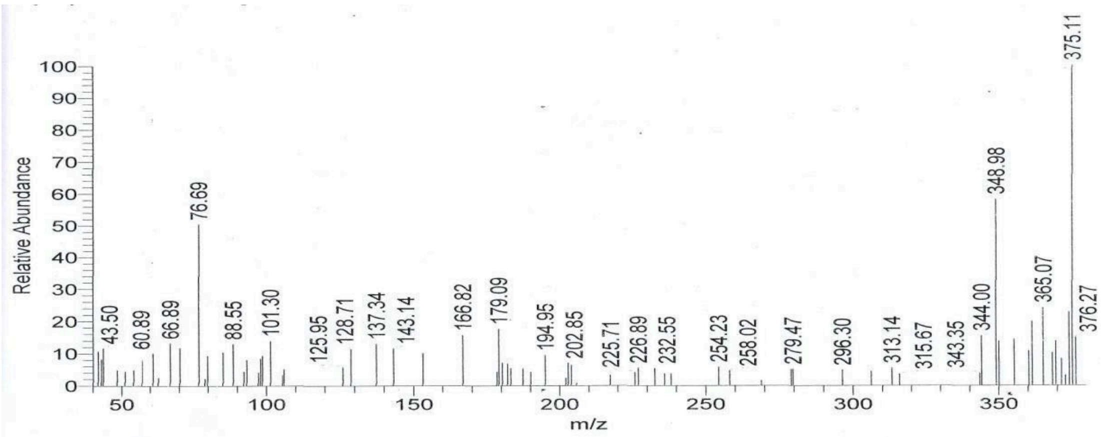

Figure S5c: Mass spectrum of compound (5a)

3-**N¹-(Acridin-9-yl) bezene-1,4-diamine(5b)**: yield (0.267gm, 79%) as a deep orange solid, m.p. >250°C. IR (KBr) cm-1 : 3380(NH2+NH) overlap, 3080(CH-Ar), 1622(C=N), 1561(C=C-Ar), 1364(C-N-Ar) , 1256(=C-N).¹H – NMR(DMSO-d6, 400MHZ),δ(ppm): 4.78 (br.s, 2H, NH2), 6.54 – 6.98 (m, 5H, 4Ar-H+ NH-Ar), 7.47 – 7.54 (m, 8H, Ar-H). EIMS, m/z (C19H15N3) calcd,285.34 [M]+; found,285.07.

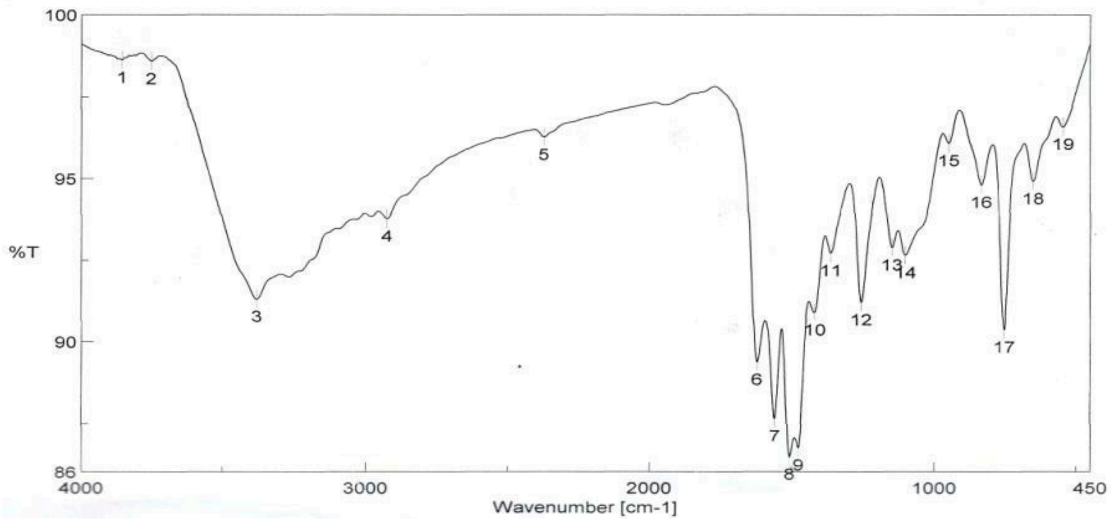

Figure S6a: IR Spectrum of compound (5b) in KBr

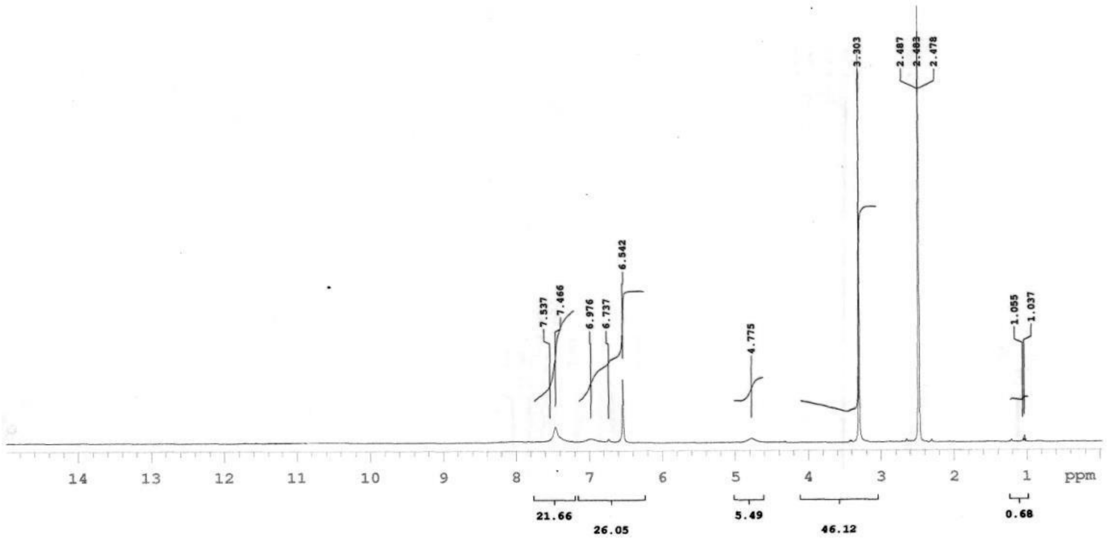

Figure S6b: ^{1}HNMR spectrum of compound 5b in DMSO

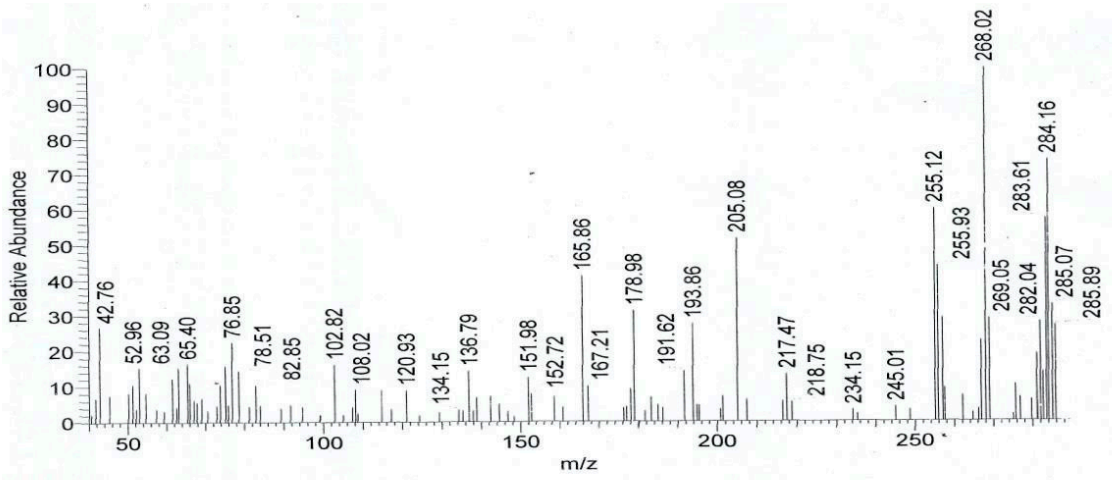

Figure S6c: Mass spectrum of compound (5b)

4- **1–(4-(4-(Acridin-9-ylamino)benzyl)phenyl)-3-phenylthiourea(6a):** yield (0.107gm, 78.5%), as a red solid, m.p. 198°C, IR (KBr) cm-1: 3427 (NH), 3024 (CH-Ar), 2918 (CH-Alkane), 1633 (C=N), 1585(C=C –Ar) , 1311(C-N , Aromatic amine), 1245(=C-N), 1110 (C=S).^{1}H – NMR (CDCl3, 300MHZ),δ(ppm): 3.53 (br.s, 2H, CH2), 7.08– 7.20 (m, 8H, Ar-H), 7.31– 7.44 (m, 5H, 5Ar-H), 7.76 – 7.85 (br.s, 1H, NH-Ar), 8.01– 8.04 (m, 8H, Ar-H), 10.76- 10.84 (br.s, 1H, -CS-HN-C6H5), 12.05 - 12.25 (br.s, 1H, -C6H4-HN-CS-). EIMS, m/z (C33H26N4S) calcd,510.65 [M]+; found,510.43.

5- **1 –(4-(Acridin-9-ylamino)phenyl)-3-phenylthiourea(6b):** yield (0.093gm, 63.33%), as a brownish red solid, m.p. >250°C. **IR** (KBr)cm-1: 3427 (NH), 3030 (CH-Ar), 1630 (C=N), 1588(C=C-Ar),1315(C-N, Ar amine),1243(=C-N),1161(C=S).**^{1}H–NMR**(DMSO-d6, 400MHZ),δ(ppm): 6.53 – 7.17 (m, 4H, Ar-H), 7.21 – 7.68 (m, 5H, Ar-H), 7.87 – 8.30 (m, 8H, Ar-H), 9.257 (br.s, 1H, NH-Ar), 10.06 (s, 1H, -CS-NH-C6H5), 10.10 (s, 1H, -C6H4-NH-CS-). EIMS, m/z (C26H20N4S) calcd,420.53 [M]+; found, 419.14.

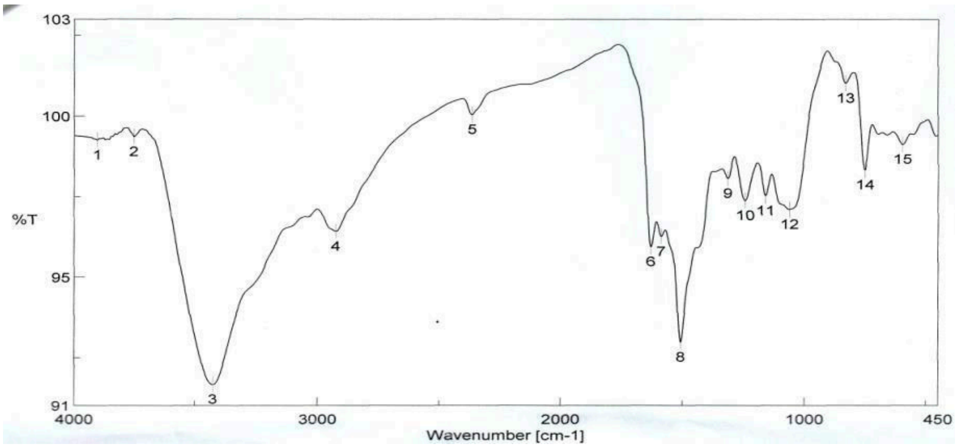

Figure S7a: IR Spectrum of compound (6b) in KBr

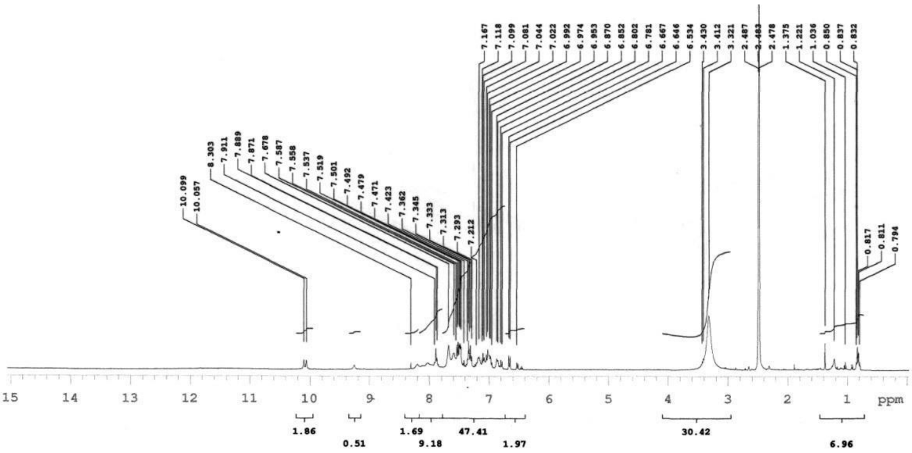

Figure S7b: ^{1}HNMR spectrum of compound (6b) in DMSO

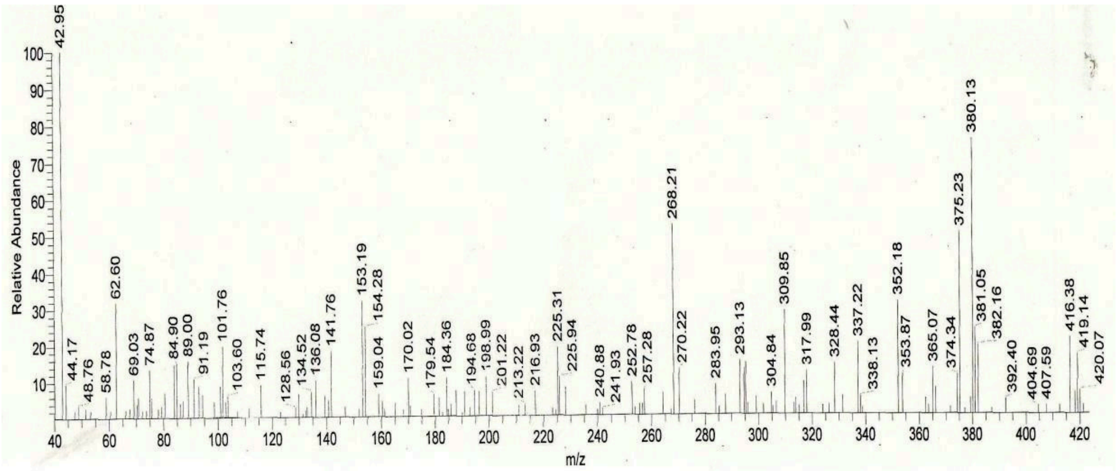

Figure S7c: Mass spectrum of compound (6b)

6- **N-(4-(4-(Acridin-9-ylamino)benzyl)phenyl)-2-nitrobenzenesulfonamide (7a):** Yield (0.072gm, 48%) as a light orange solid, m.p. 172°C, IR (KBr) cm-1: 3431 (NH), 3038 (CH – Ar), 2924 (CH – Alkane), 1623 (C=N), 1511(C=C – Ar),1258 (C-N Aromatic amine), 1160 (=C-N), 1509, 1346(N-O, asym, sym). ^{1}H – NMR (CDCl3, 300MHZ), δ(ppm): 2.90 (s, 2H, CH2), 6.75 – 7.11 (m, 9H, 8Ar-H+NH-Ar), 7.56 – 8.03 (m, 12H, Ar-H), 8.20 – 8.38 (m, 1H, HN-SO2). EIMS, m/z (C32H24N4SO4) calcd,560.62[M]+; found,560.07.

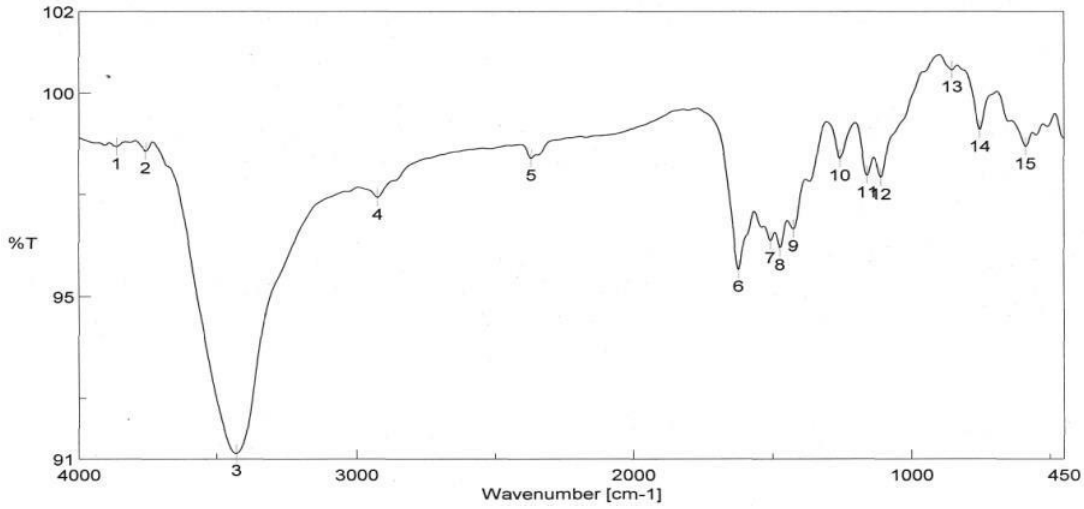

Figure S8a: IR Spectrum of compound (7a) in KBr

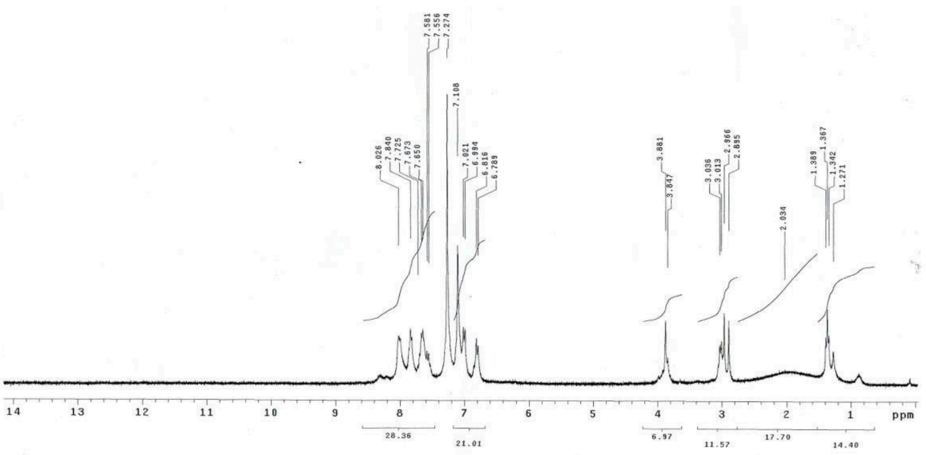

FigureS8b: ^{1}HNMR Spectrum of compound (7a) in chloroform

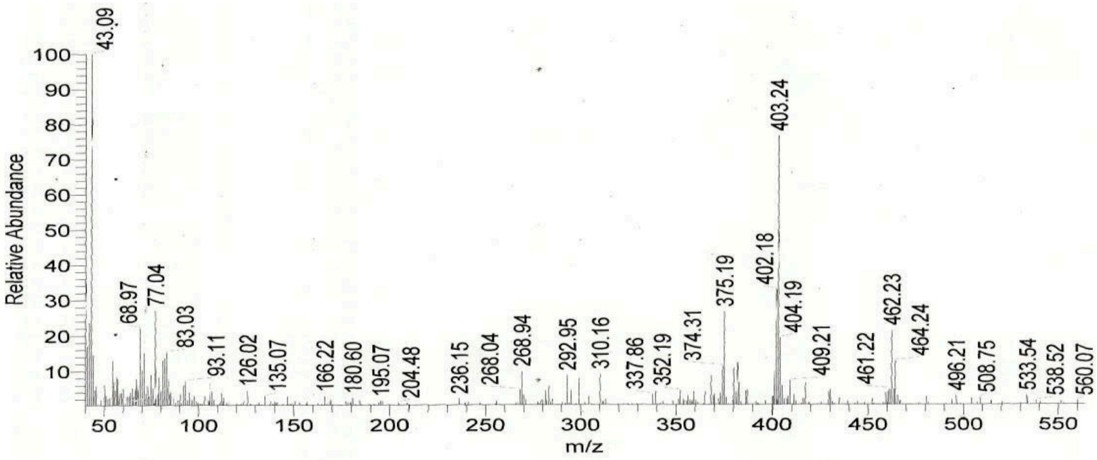

Figure S8c: Mass spectrum of compound (7a)

7- **N-(4-(4-(Acridin-9-ylamino)benzyl)phenyl)-4-methylbenzenesulfonamide(7b):**Yield (0.08gm, 57%) as a yellow solid, m.p.>250°C. IR (KBr) cm-1: 3426 (NH), 3028 (CH-Ar), 2923 (CH-Alkane), 1620 (C=N), 1592 (C=C-Ar), 1418 (C-N-Ar amine), 1334, 1092(S=O,asym,sym),1157(=C-N).^{1}H – NMR (CDCl3, 300MHZ), δ(ppm): 2.39 (br.s, 3H, CH3), 2.89 (br.s, 2H, CH2), 6.26 – 6 .40 (br.s, 1H, NH-Ar), 6.96– 7.24 (m, 8H, Ar-H), 7.62 – 7.64 (m, 12H, Ar-H), 7.98 – 8.05 (br.s, H, HN- SO2). EIMS, m/z (C33H27N3SO2) calcd,529.65 [M]+; found,529.34. N-((tetrahydrofuran-2-yl)methyl)-11H-indolo[3,2-c]quinolin-6- amine(5k)

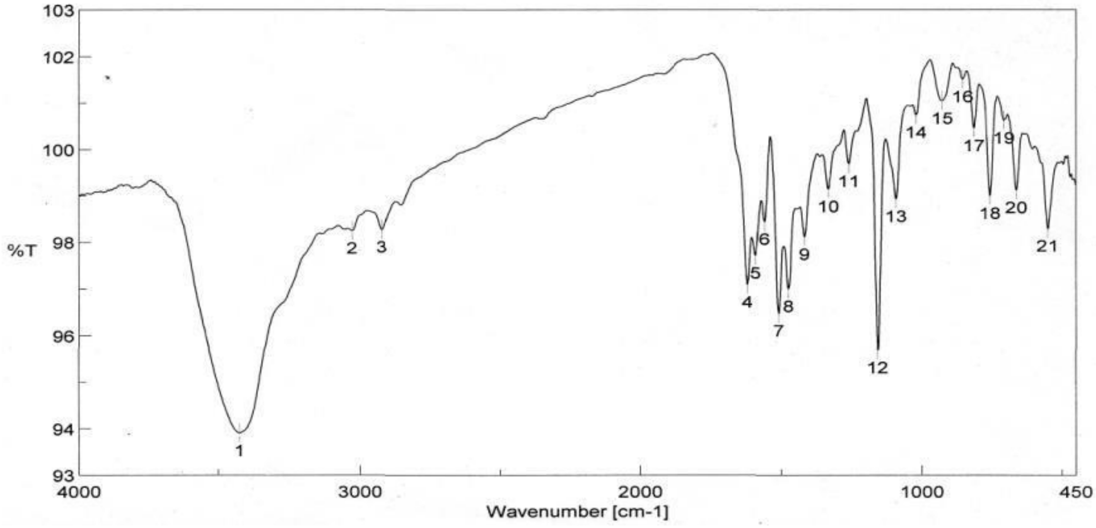

Figure S9a: IR Spectrum of compound (7b) in KBr

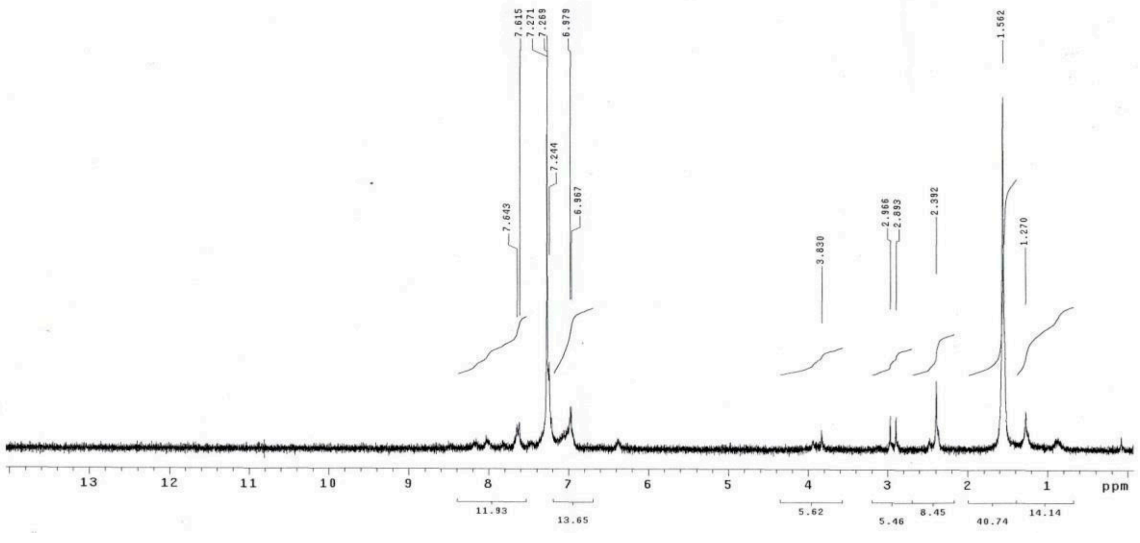

Figure S9b: ^{1}HNMR Spectrum of compound (7b) in chloroform

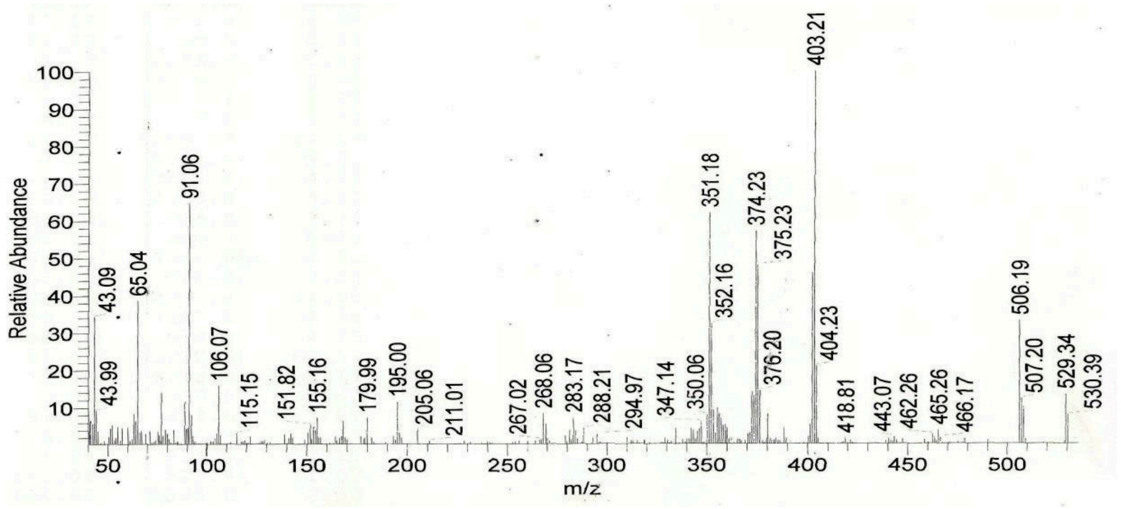

Figure S9c: Mass spectrum of compound (7b)

8 - N-(4-(N-(4-(4-(Acridin-9-ylamino)benzyl)phenyl)sulfamoyl)phenyl)acetamide(7c):

yield (0.043gm, 28.67%) as a dark yellow solid, m.p. >250°C. **IR** (KBr) cm-1:3434 (NH), 3037 (CH-Ar), 2922 (CH-Alkane), 1623 (C=N), 1511 (C=O), 1473(C=C-Ar), 1420, 1157(S=O asym, sym), 1260 (C-N, Ar amine), 1157(=C-N).**^{1}H NMR** (CDCl3, 300MHZ),δ(ppm): 2.89 (m, 2H, CH2), 3.60 (br.s, H, NH), 3.84 (s, 3H, CH3), 6.61 – 6.65 (m, 4H, Ar-H) , 6.81 – 6.85 (m, 4H, Ar-H), 6.95 – 7.11 (m, 4H, 4Ar-H), 7.63– 7.68 (m, 8H, Ar-H), 8.00 – 8.03 (m, 2H, HN-SO2 +HN-CO). EIMS, m/z (C34H28N4SO3) calcd,572.68[M]+; found,572.39.

(C34H28N4SO3) calcd,572.68[M]+; found,572.39.

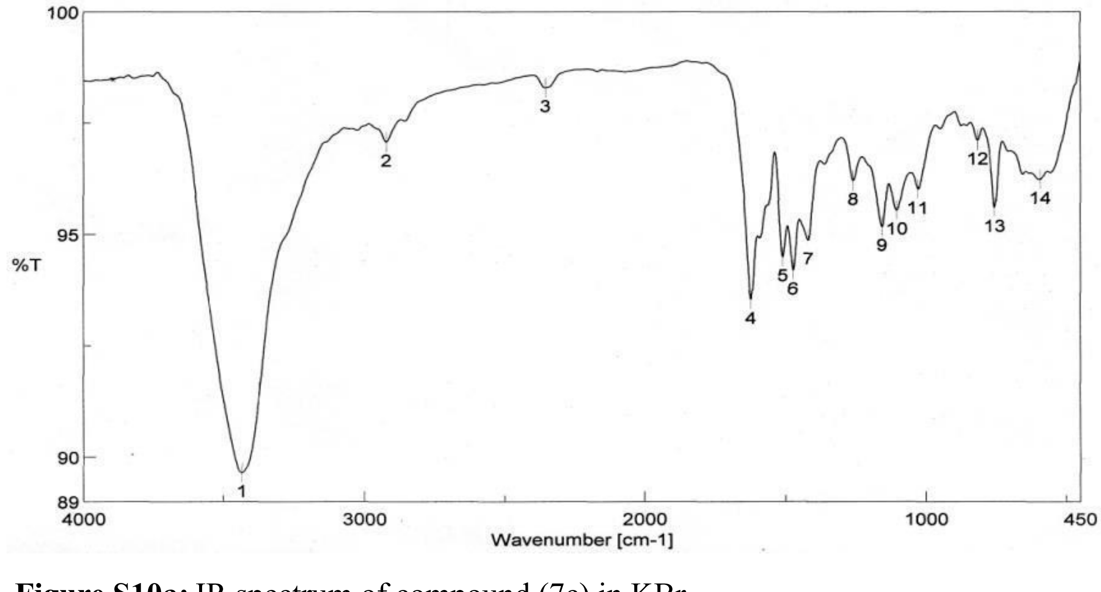

Figure S10a: IR spectrum of compound (7c) in KBr

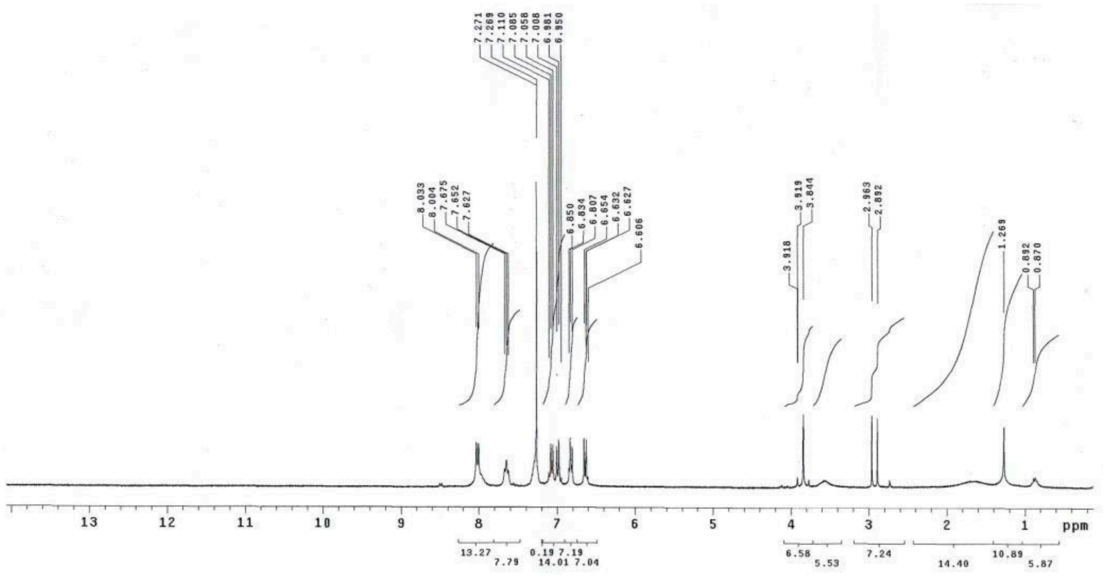

Figure S10b: ^{1}HNMR Spectrum of compound (7c) in chloroform

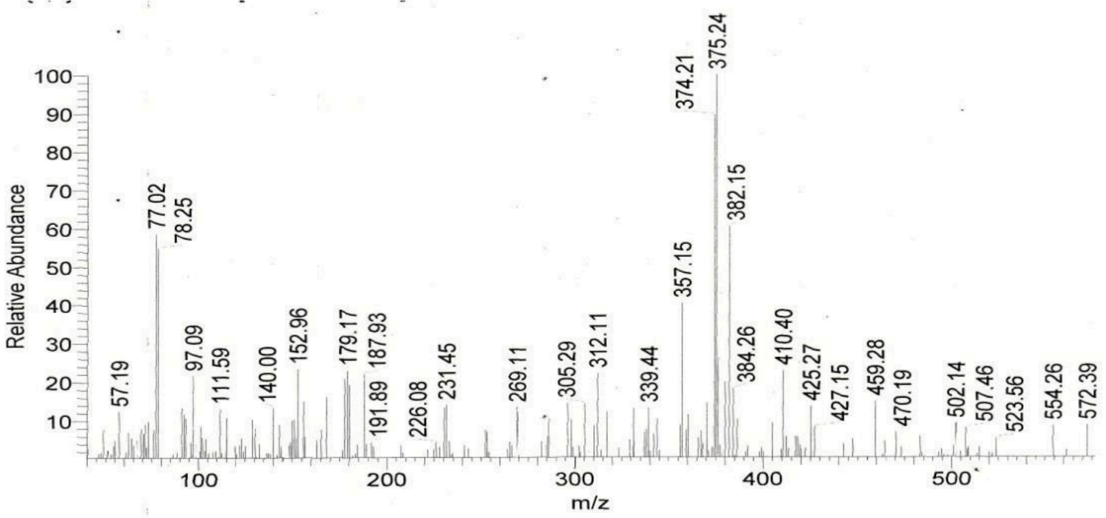

Figure S10c: Mass spectrum of compound (7c)

9-N-(4-(4-(Acridine-9-ylamino) benzyl) phenyl)-4-(phenyldiazenyl)benzenesulfonamide(7d)

yield (0.125gm, 75%), as an orange solid, m.p.>250°C. **IR** (KBr) cm-1: 3433 (NH), 3040 (CH-Ar), 2925 (CH-Alk), 1631 (C=N), 1590 (C=C-Ar), 1512 (N=N), 1439, 1119 (S=O, asym, sym), 1342 (C-N, Ar amine), 1165 (=C-N).**^{1}H – NMR** (CDCl3, 300MHZ),δ(ppm):2.89(s, 2H, CH2), 6.58 – 6.65 (br.s, 1H, NH-Ar), 7.00 – 7.07 (m, 8H, Ar-H), 7.27 – 7.55 (m, 4H, Ar-H), 7.74 – 8.05 (m, 8H, Ar-H), 8.25 – 8.49(m, 5H, Ar-H), 10.80 – 10.90 (brs, 1H, HN-SO2) . EIMS, m/z (C38H29N5SO2) calcd,619.73[M]+; found,619.34.

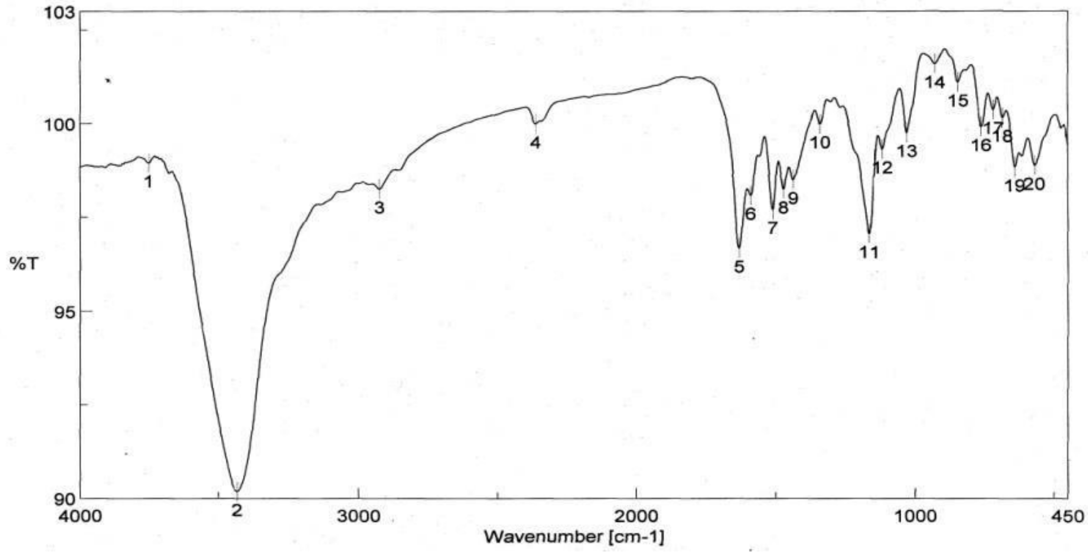

FigureS11a: IR Spectrum of compound (7d) in KBr

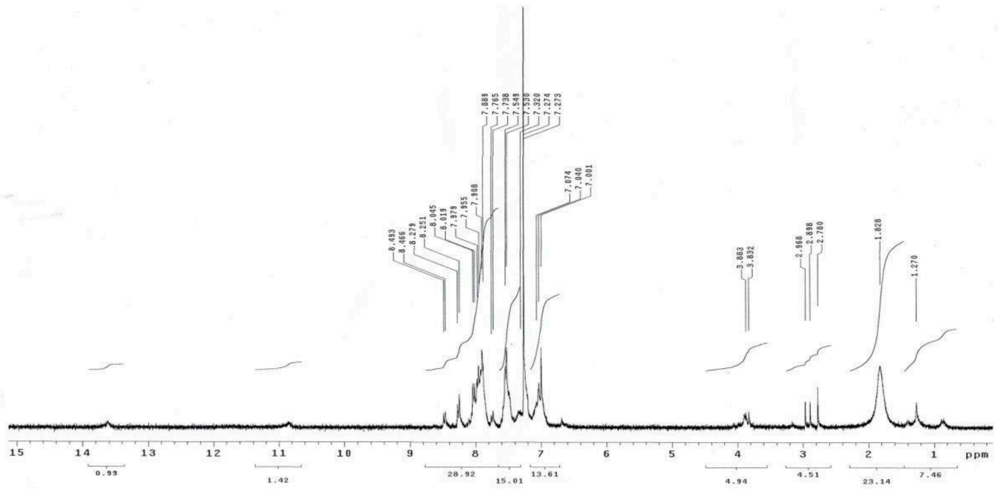

Figure S11b: ^{1}HNMR of compound (7d) in chloroform

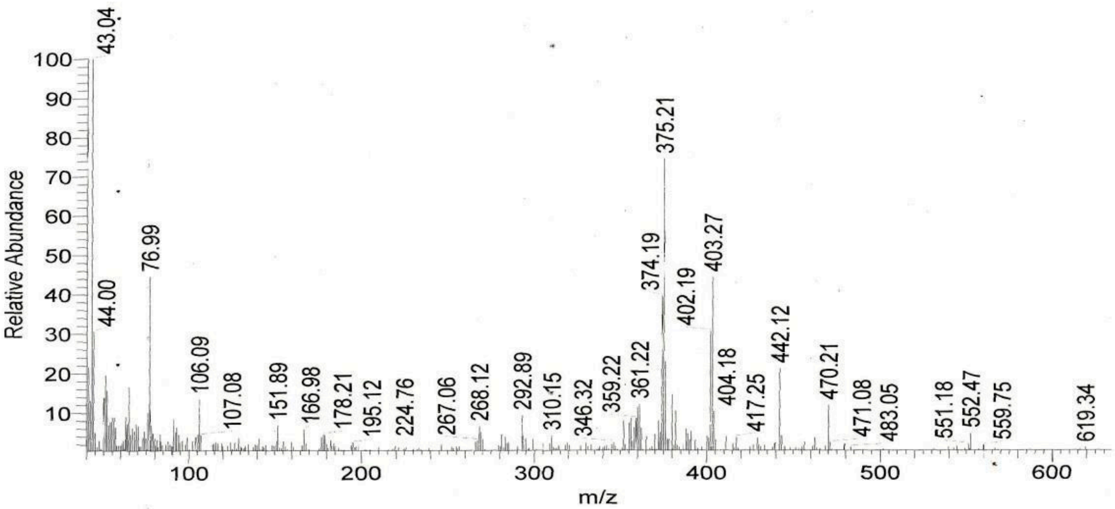

Figure S11c: Mass spectrum of compound (7d)

10 - N-(4-(4-(Acridine-9-ylamino)benzyl)phenyl)-2,4,6-triisopropylbenzenesulfonamide(7e)

yield (0.07gm, 78.5%), as an olive solid, m.p. 168°C. **IR** (KBr) cm-1: 3418 (NH), 3033 (CH-Ar), 2958 (CH-Alkane), 1672 (C=N), 1628 (C=C), 1472, 1158 (S=O, asym, sym), 1339 (C-N,Ar amine), 1262 (=C-N).**^{1}H – NMR** (CDCl3, 300MHZ),δ(ppm): 1.25 – 1.35(br.s, 18H, 6CH3), 2.89 (m, 2H, CH2), 3.86-3.92 (m, 3H, CH), 6.65 – 7.23 (m, 11H, 10Ar-H + NH-Ar), 7.35 – 8.42 (m, 8H, Ar- H), 9.70 – 9.80 (br.s, 1H, HN-SO2). EIMS, m/z (C41H43N3SO2) calcd,641.86[M]+; found,641.71.

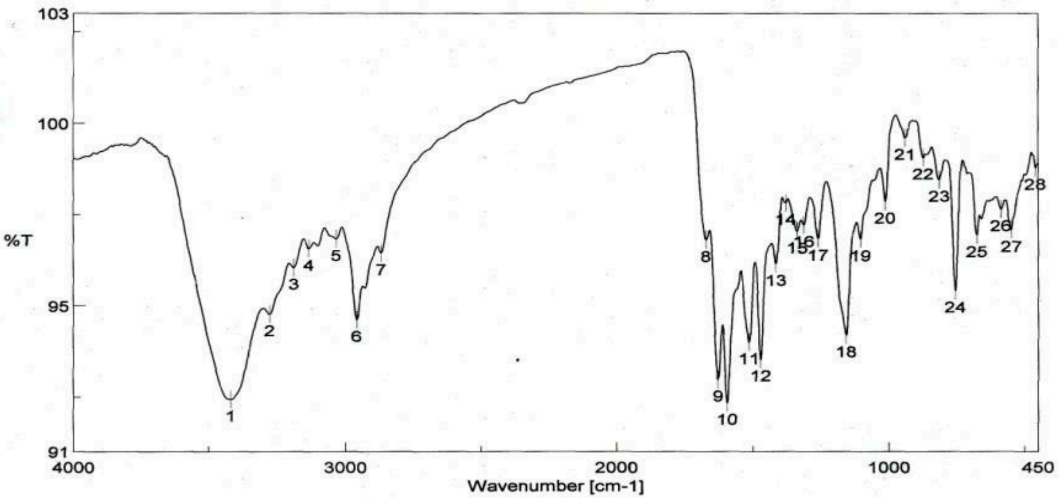

Figure S12a: IR Spectrum of compound (7e) in KBr

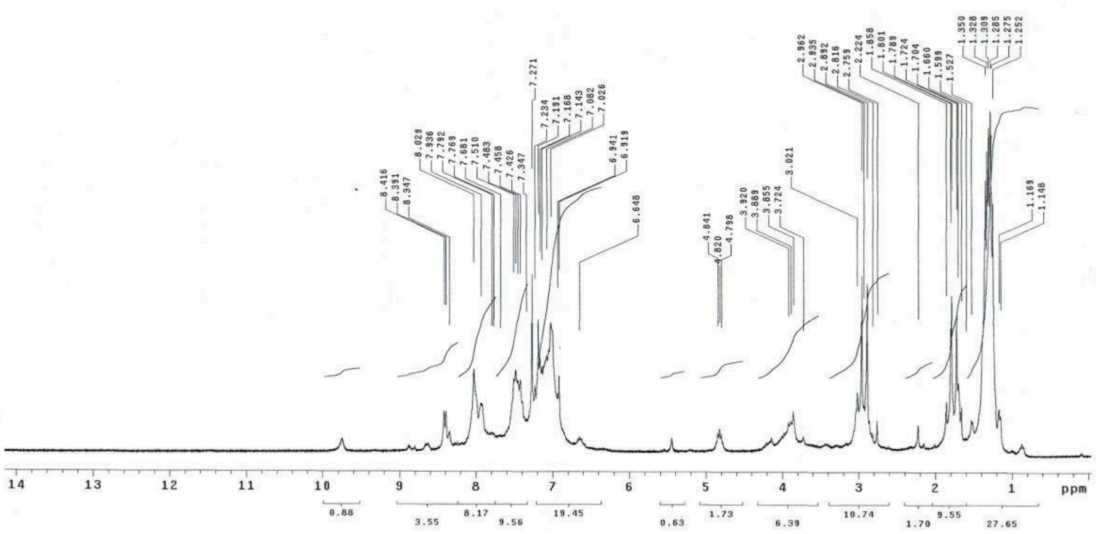

Figure S12b: ^{1}HNMR spectrum of compound 7e in chloroform

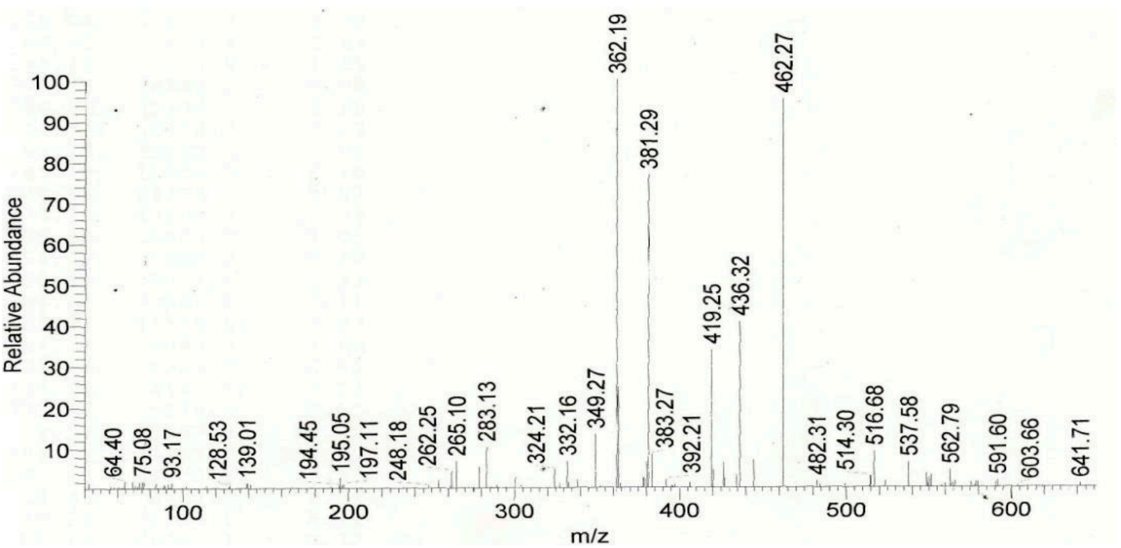

Figure S12c: Mass spectrum of compound (7e)

11- **N-(4-(Acridin-9-ylamino)phenyl)-2-nitrobenzenesulfonamide(8a):** yield (0.078gm, 47%), as abrownishsolid, m.p.>250°C. **IR** (KBr) cm-1: 3423 (NH), 3112 (CH-Ar), 1628 (C=N), 1584 (C=C-Ar), 1505 , 1288 (N-O asym, sym), 1366,1104 (S=O, asym, sym), 1254 (C-N).**^{1}H – NMR** (DMSO-d6, 400MHZ), δ(ppm):[6.54 – 6.72 (m, 4H, Ar-H), 7.01 – 7.43 (m, 8H, Ar-H), 7.81 – 7.96 (m, 4H, Ar-H),10.22 (br.s, 1H, NH-Ar), 10.84 (br.s, 1H, NH-SO2). EIMS, m/z (C25H18N4SO4) calcd,470.50[M]+; found, 470.05.

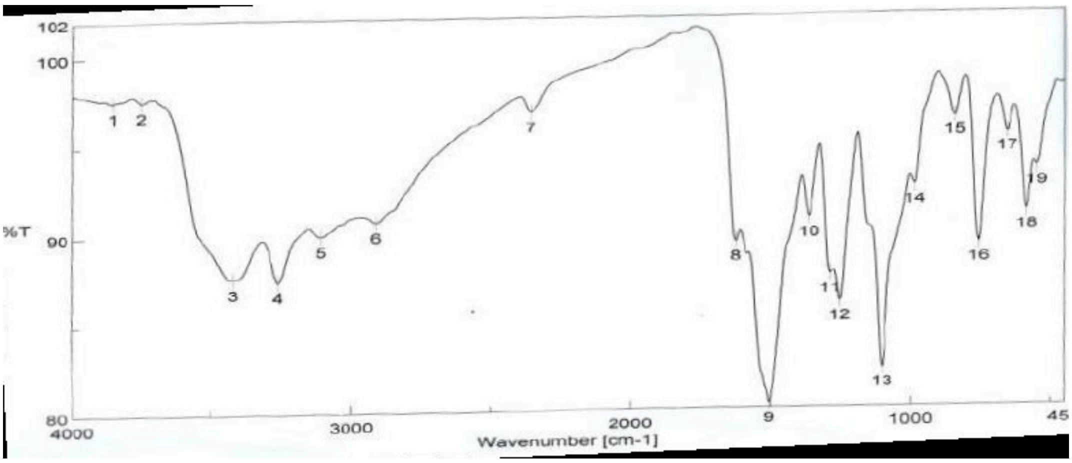

Figure S13a: IR Spectrum of compound (8a) in KBr

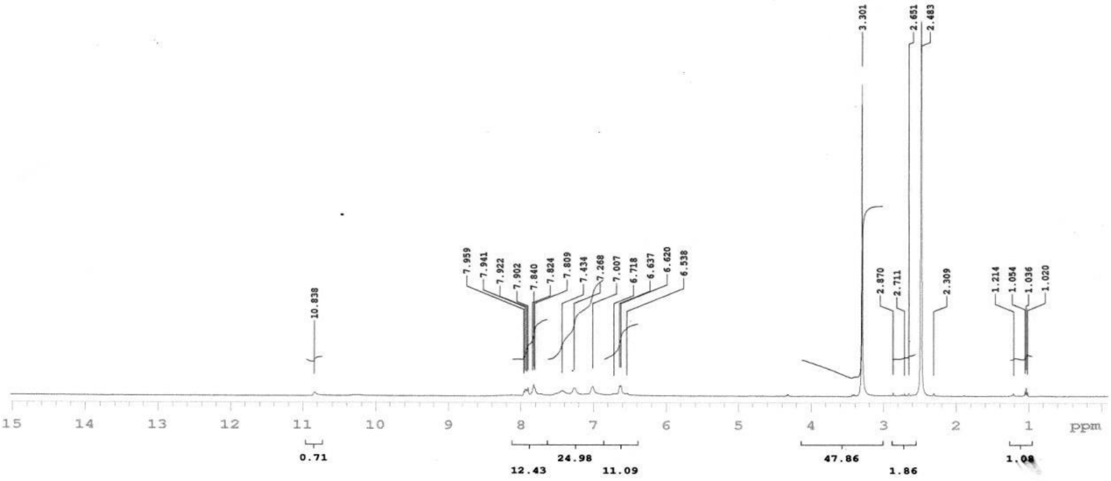

Figure S13b: ^{1}HNMR spectrum of compound 8a in DMSO

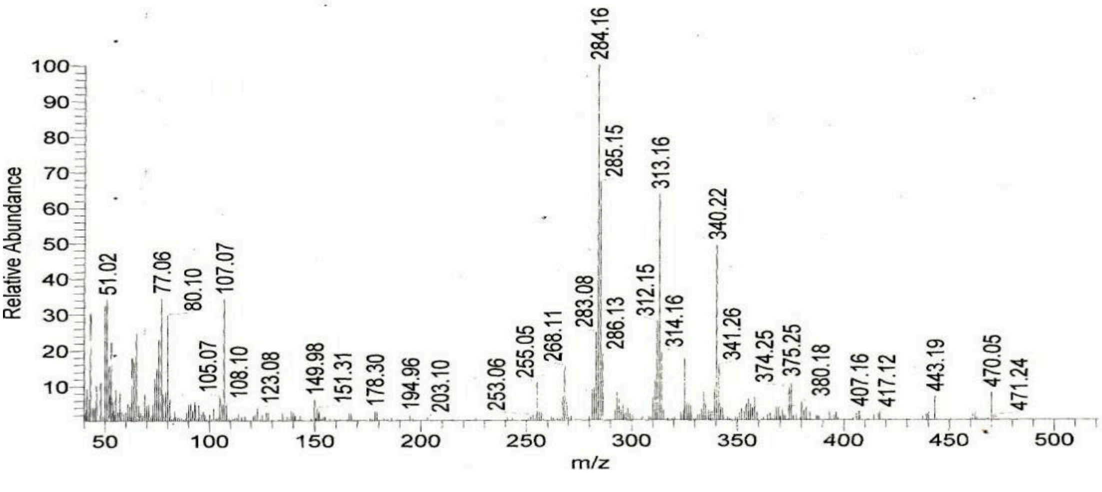

Figure S13c: Mass spectrum of compound (8a)

12-N-(4-(Acridin-9-ylamino)phenyl)-4-methylbenzenesulfonamide(8b):

yield (0.096gm, 62%), as a light brown solid, m.p. >250°C. **IR** (KBr)cm-1: 3324(NH), 3089 (CH-Ar), 1619 (C=N), 1589 (C=C-Ar), 1328, 1154 (S=O, asym, sym), 1258 (C-N).^{1}**H – NMR** (DMSO-d6, 400MHZ),δ(ppm): 2.35 (s, 3H,CH3), 6.57 – 6.99 (m, 8H, Ar-H), 7.25 – 7.60 (m, 8H, Ar-H), 8.03(br.s, 1H, NH- Ar), 9.80 (br.s, 1H, NH-SO2). EIMS, m/z (C26H21N3SO2) calcd,439.53 [M]+;found,439.19.

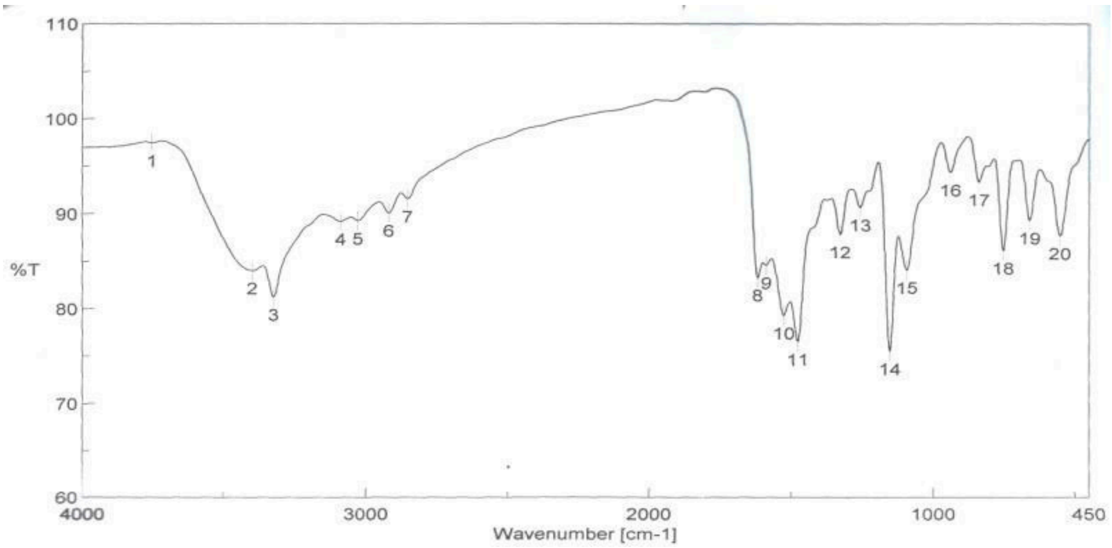

Figure S14 a: IR Spectrum of compound (8b) in KBr

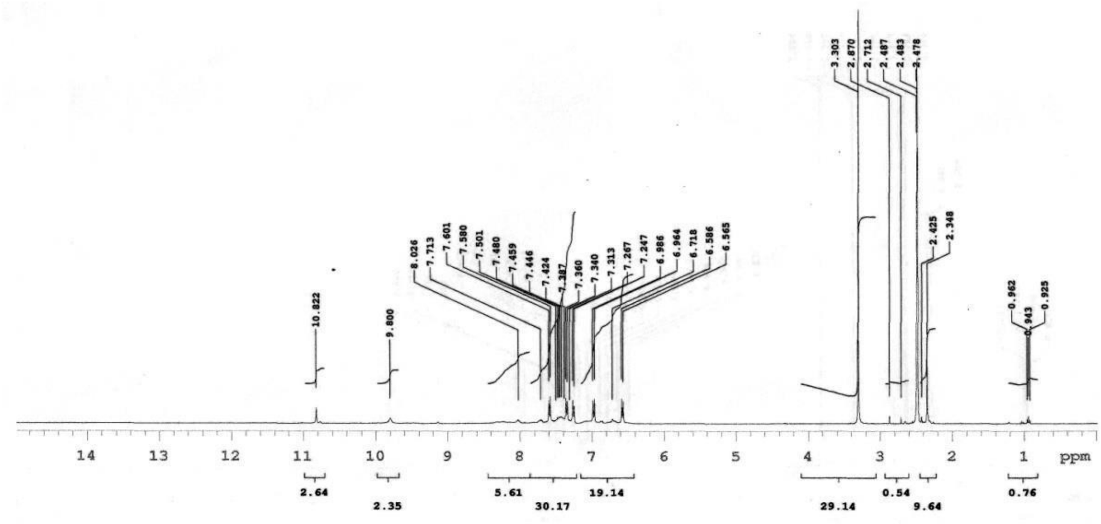

Figure S14 b: ^{1}HNMR spectrum of compound 8b in DMSO

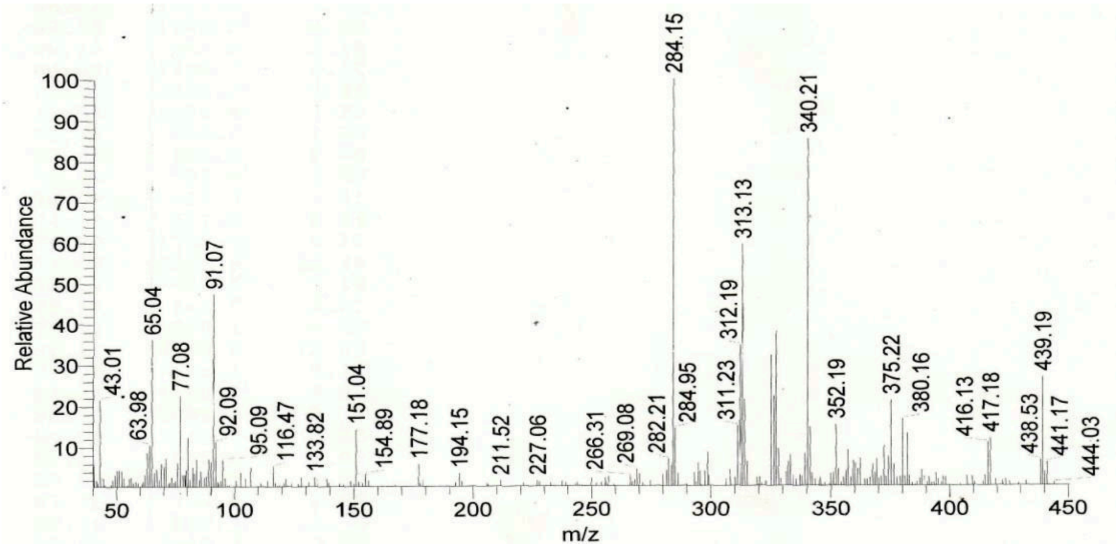

Figure S14c: Mass spectrum of compound (8b)

13-N-(4-(Acridin-9-ylamino)phenyl)-4(phenyldiazenyl)benzenesulfonmide(8c): yield (0.158gm, 84.98%), as a brown solid, m.p. >250°C. IR (KBr)cm-1: 3420(NH), 3250 (CH-Ar), 1626 (C=N), 1510(C=C-Ar), 1470 (N=N), 1335, 1035 (S=O, asym, sym), 1158(C-N).^{1}H – NMR (DMSO-d6, 400MHZ), δ(ppm): 6.37 – 6.94 (m, 4H, Ar-H), 7.00 –8.22 (m, 17H, Ar-H), 10.16 (br. s, H, NH-Ar), 10.41(br. s, H, NH-SO2). EIMS, m/z (C31H23N5SO2) calcd,529.61[M]+; found,529.21.

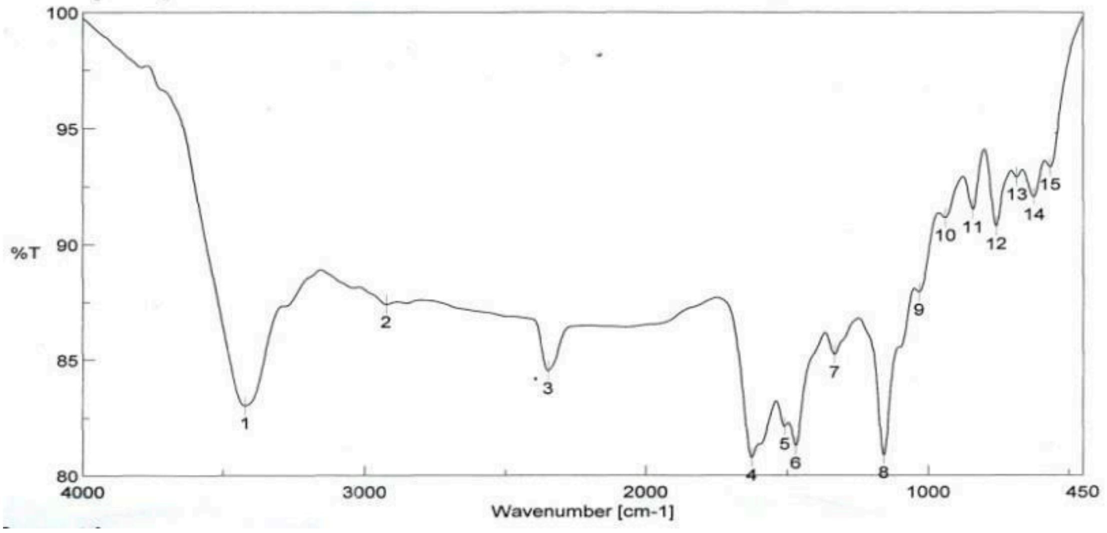

Figure S15a: IR Spectrum of compound (8c) in KBr

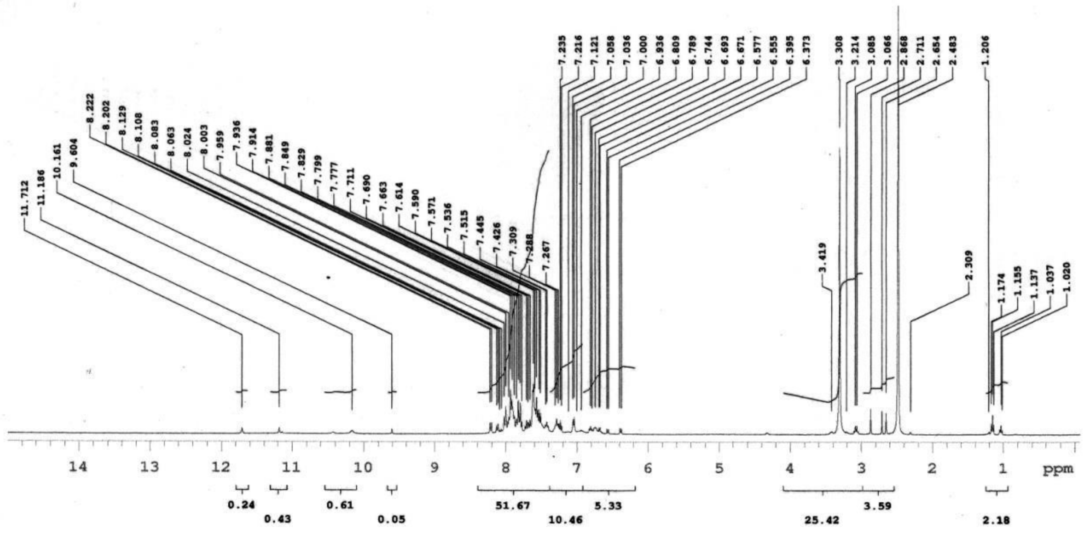

Figure S15 b: ^{1}HNMR spectrum of compound 8c in DMSO

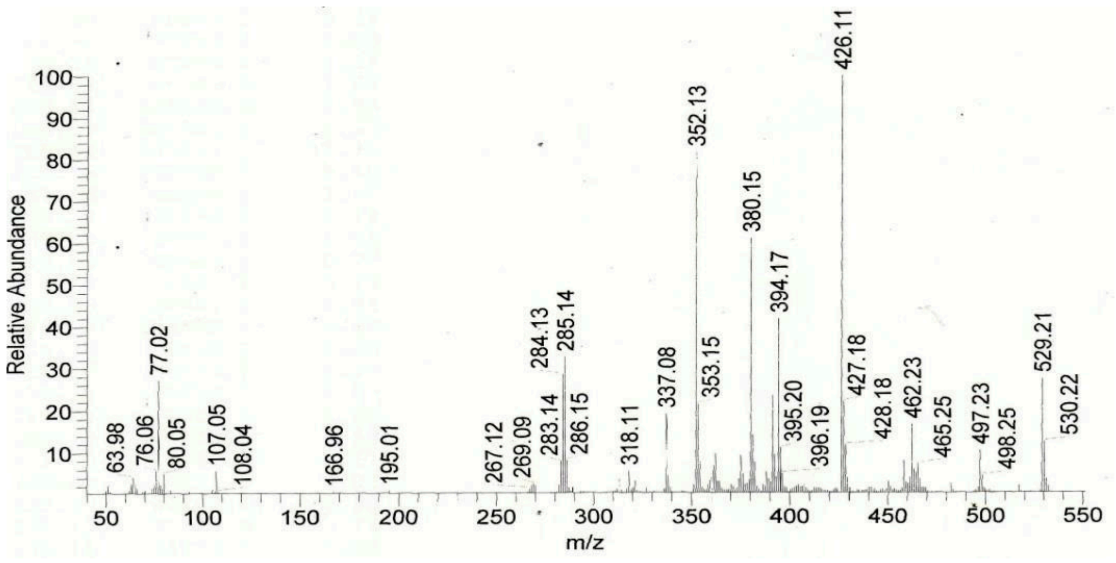

Figure S15c: Mass spectrum of compound (8c)

14-N-(4-(Acridin-9-ylamino) phenyl)-

2,4,6triisopropylbenzenesulfonamide(8d): yield (0.078gm, 40%), as a light brown solid, m.p. >250°C. **IR** (KBr)cm-1: 3425 (NH), 3250 (CH-Ar),2958(CH- Alkane), 1619 (C=N), 1563(C=C), 1369, 1152 (S=O, asym, sym), 1259 (C-N). **^{1}H – NMR** (DMSO-d6, 400MHZ), δ(ppm): 2.43 (s, 18H, 6CH3), 2.87 – 2.91(m, 3H, 3CH), 6.59 – 6.98 (m, 4H, Ar-H), 7.20 – 7.26 (m, 2H, Ar-H), 7.41 –8.01 (m, 8H, Ar-H), 8.30(br. s, 1H, NH-Ar), 9.82 (br. s, 1H, NH-SO2). EIMS, m/z (C43H37N3SO2) calcd,551.74[M]+; found,551.42.

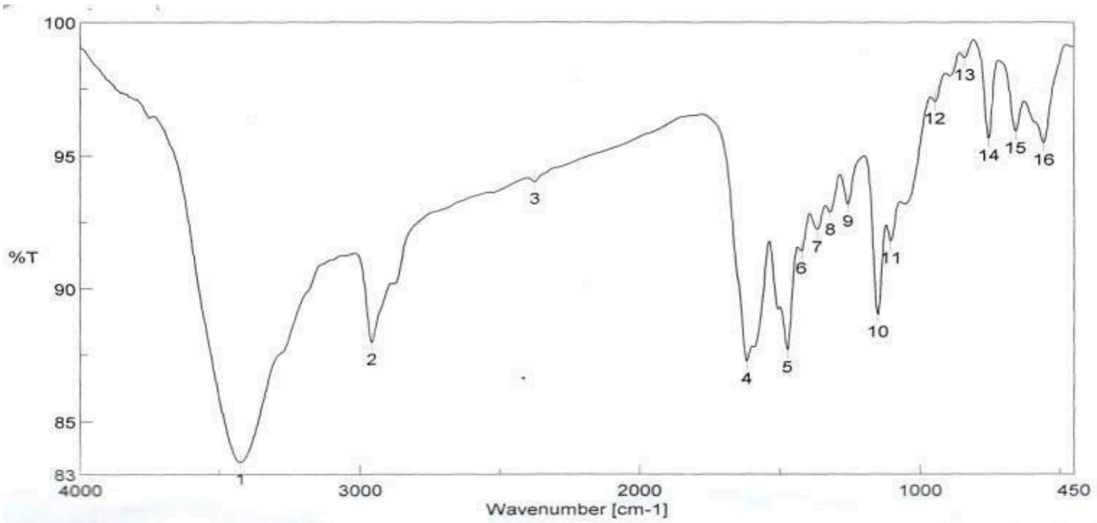

Figure S16a: IR Spectrum of compound (8d) in KBr

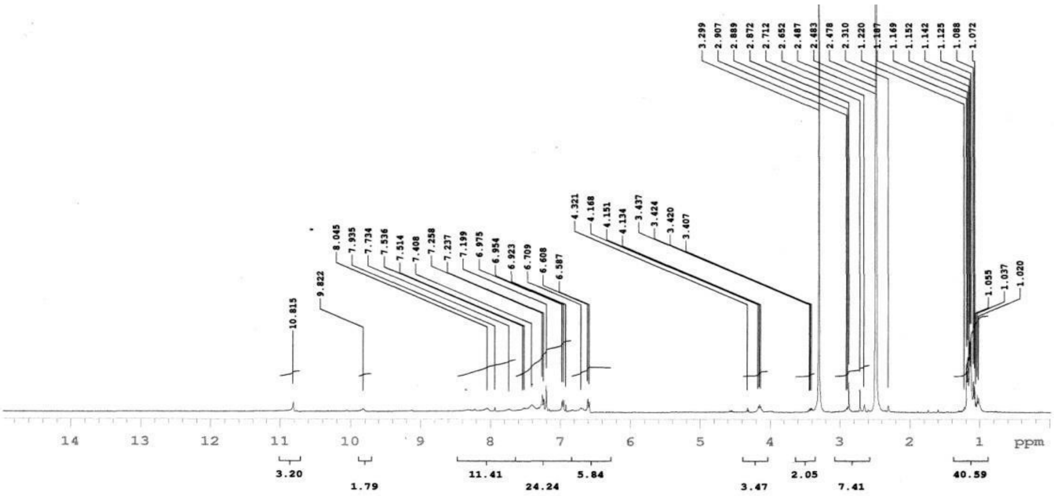

Figure S16 b: ^{1}HNMR spectrum of compound 8d in DMSO

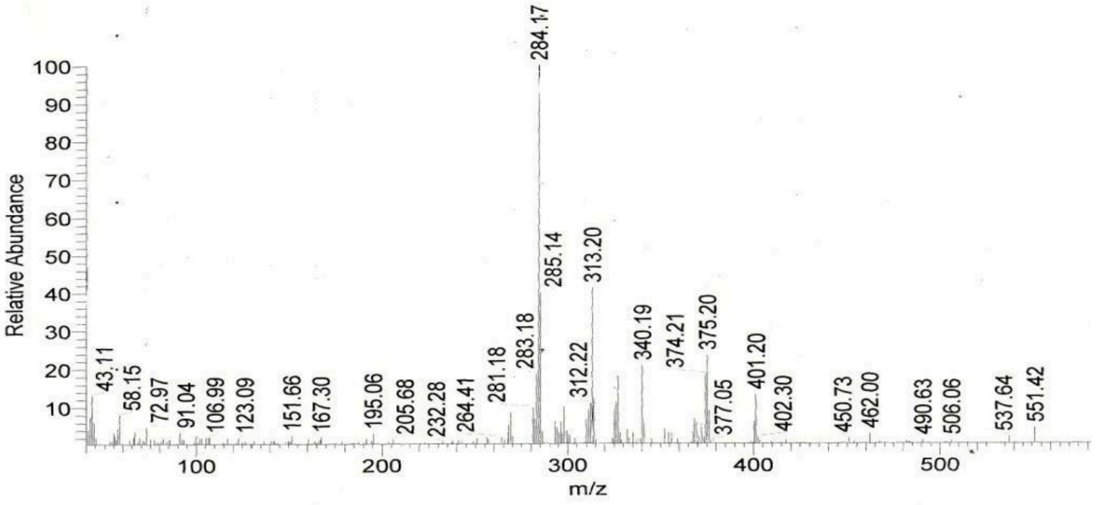

Figure S16 c: Mass spectrum of compound (8d)
